# Statistical inference for the generalized exponential distribution using ordered lower k-record ranked set sampling with random sample sizes

**DOI:** 10.1038/s41598-025-01995-z

**Published:** 2025-05-30

**Authors:** Haidy A. Newer

**Affiliations:** Department of Mathematics, Faculty of Education, Ain Shams University, Cairo, 11511 Egypt

**Keywords:** Ordered lower k-records moving extremes ranked set sampling, Empirical Bayes inference, Generalized exponential model, Pivotal prediction method, Monte Carlo simulation, Materials science, Mathematics and computing, Physics

## Abstract

This article presents an innovative sampling strategy, ordered moving extremes lower *k*-record ranked set sampling, designed to enhance parameter estimation and prediction for the generalized exponential distribution. By incorporating *k*-record values with random sample sizes, we develop maximum likelihood estimation, classical Bayes estimation, and empirical Bayes estimators, leveraging informative priors under balanced loss functions, including balanced squared error and balanced linear exponential. Additionally, we utilize the pivotal prediction method to construct prediction intervals for future observations under double type-II censoring. Extensive simulation studies demonstrate that our approach significantly improves estimation accuracy by achieving lower mean squared errors and reduced bias compared to conventional methods. The efficacy of the proposed sampling method is further validated through its application to real-world medical datasets, showcasing its practical utility in enhancing statistical inferences for lifetime data analysis. The key findings highlight that ordered moving extremes lower *k*-record ranked set sampling effectively balances efficiency and accuracy, making it particularly well-suited for reliability studies and survival analysis.

## Introduction

In reliability analysis and lifetime testing, the high costs and extensive time required to measure key characteristics demand efficient sampling methods. This challenge is particularly pronounced in fields such as reliability engineering, environmental studies, and medical research, where collecting data-especially on extreme events-can be resource-intensive and time-consuming. Ranked set sampling (RSS) provides a cost-effective alternative by strategically selecting samples to improve observational efficiency and estimation precision. First proposed by McIntyre^1^, RSS enhances the accuracy of the sample mean as an estimator of the population mean, making it a valuable tool in statistical sampling. Its versatility is evident in its broad applications, as demonstrated by works such as Ali et al.^2^, Bhushan and Kumar^3–6^, Kotb et al.^7^, Mohie El-Din et al.^8–11^, Newer et al.^12–14^, Qian et al.^15^, and Stokes^16^. Recent advancements, notably by Bhushan and Kumar^3–6^, have extended RSS to complex data structures and computational frameworks, reinforcing its relevance in modern statistical practice. Notably,^17^ highlighted RSS’s adaptability to exponential-based distributions, directly aligning with our framework based on the generalized exponential distribution. Supporting this,^18,19^ showcased RSS’s effectiveness in parameter estimation for complex distributions, underscoring the value of advanced sampling strategies in our approach. Additionally,^20–22^ emphasized RSS’s role in stress-strength reliability estimation, complementing our focus on reliability analysis. Further enriching this context,^23,24^ explored advanced RSS variants to enhance reliability discussions, providing a broader foundation for our methodological contributions. This paper explores RSS applications in reliability analysis and lifetime testing, emphasizing its potential to optimize resource allocation and enhance inference in high-cost data collection scenarios. To address ranking errors while retaining RSS efficiency, Al-Odat and Al-Saleh^25^ developed moving extremes ranked set sampling (MERSS), an innovative modification. MERSS employs a two-phase process: in the first phase, $m_{1}$ simple random samples (SRS) of increasing sizes-$1, 2, \ldots , m_{1}$-are drawn, ranked using cost-effective methods (e.g., visual inspection) without direct measurement, and the maximum observation from each set is measured. In the second phase, the process repeats with $m_{2}$ sets (sizes $1, 2, \ldots , m_{2}$), focusing on measuring the minimum observation from each set. The result is a MERSS sample of size $n = m_{1} + m_{2}$. Recent studies, such as Bhushan and Kumar^5,6^, have advanced RSS by integrating computational techniques, enhancing its utility for large-scale data applications and highlighting its adaptability to contemporary research demands.

Among ordered data types, record statistics have gained prominence for analyzing extreme phenomena. In a sequence of random variables (RVs), upper (lower) $k$-record statistics denote the subsequence of the $k$th largest (smallest) values, with standard records corresponding to $k=1$. These statistics are widely applied in fields like industrial stress testing, finance, weather analysis, and sports. For a sequence $\{R_{a}, a \ge 1\}$ of independent and identically distributed (iid) RVs, an upper record $R_{i}$ satisfies $R_{j} < R_{i}$ for all $j > i$, and a lower record satisfies $R_{j} > R_{i}$ for all $j > i$, with $R_{1}$ as the initial upper and lower record. In the continuous case, lower $k$th record values and inter-$k$-record times (the number of observations required to obtain a new *k*-record) are defined as follows: for a fixed $k$, the sequence $\{T_{a,k}, a \ge 1\}$ of $k$th lower record times is given by $T_{1,k} = 1$, and for $a \ge 1$, $T_{a+1,k} = \min \{j> T_{a,k}: R_{k:T_{a,k}+k-1} > R_{k:j+k-1}\},$ where $R_{j:a}$ is the $j$th order statistic of $(R_{1}, R_{2}, \dots , R_{a})$. The sequence $\{\omega _{a(k)}, a \ge 1\} = \{R_{k:T_{a,k}+k-1}, a \ge 1\}$ represents the $k$th lower record values, and inter-$k$-record times are $\Delta _{(k)} = T_{a+1,k} - T_{a,k}$ (see^26^ for details).

A major challenge in reliability analysis is assessing product failure times under normal conditions, where testing highly reliable products with complete samples is impractical due to cost and time constraints. Double type-II censoring addresses this by censoring data at both ends of a sample-for example, in a sample of $n$ items, the first $r$ items are left-censored, and the last $s$ are right-censored ($1< r< s < n$)-focusing on the most informative middle portion. This method excels in parameter estimation and prediction for reliability studies, offering practical advantages over type-I and hybrid censoring schemes (see^27,28^).

Statistical inference aims to derive population insights from samples, encompassing estimation, hypothesis testing, and prediction. In fields like industrial quality control and survival analysis, predicting future observations (e.g., failure times) is vital. Prediction methods include interval and point approaches, with significant contributions from Barakat et al.^29,30^ on exponential lifetimes using generalized order statistics, and Barakat and Newer^31^ on exact intervals for exponential and Pareto lifetimes via ordered RSS (ORSS).

Despite recent advancements, a significant gap remains in applying empirical Bayes estimation and pivotal prediction to the generalized exponential distribution (GED) under double type-II censored samples, particularly using ordered moving extremes lower $k$-record RSS (OMELRRSS) with independent and non-identically distributed (INID) RVs. This study aims to bridge this gap through two key objectives: (1) Introducing innovative inference methods for parameter estimation and prediction under OMELRRSS, utilizing random sample sizes to enhance accuracy, and (2) Developing empirical Bayes estimators for GED by incorporating informative priors and balanced loss functions-both squared error (symmetric) and linear exponential (LINEX, asymmetric)-to ensure robust estimation. Additionally, we construct prediction intervals for future observations under both fixed and random sample sizes, supported by explicit pivotal statistic models.

The paper is organized as follows: Section “Model description” presents the OMELRRSS-based lifetime model for INID random variables, suitable for complex data and censoring. Section “Maximum likelihood estimation” derives maximum likelihood estimators (MLEs) for model parameters. Section “Empirical Bayes estimation” estimates hyperparameters and derives empirical Bayes estimators. Section “Exact prediction intervals based on lower OMELRRSS” computes the pivotal variable’s distribution for random sample sizes. Section “MC-simulation algorithm” evaluates the methods via Monte Carlo simulation, and Section “Data analysis” applies them to real-world datasets. Section “Concluding remarks” concludes with findings and future research directions.

## Model description

The GED has been highly valued in reliability studies and lifetime testing experiments due to its mathematical simplicity and ease of manipulation. Its ability to provide closed-form solutions for key statistical quantities makes it a preferred choice for addressing a wide range of problems in these domains. In this context, we assume that the random variable $W$, representing the lifetime of a unit under lower $k$-records, follows the GED with shape parameter $\theta > 0$ and scale parameter $\lambda > 0$, denoted as $W \sim \text {GED}(\theta , \lambda )$. The probability density function (PDF) and cumulative distribution function (CDF) of $W$ are given by:

$$\begin{aligned}g(\omega ) = \theta \lambda \exp (-\lambda \omega ) \left( 1 - \exp (-\lambda \omega )\right) ^{\theta - 1}, \quad \omega > 0,\end{aligned}$$

$$\begin{aligned}G(\omega ) = \left( 1 - \exp (-\lambda \omega )\right) ^\theta , \quad \omega > 0.\end{aligned}$$

**Fig. 1:**
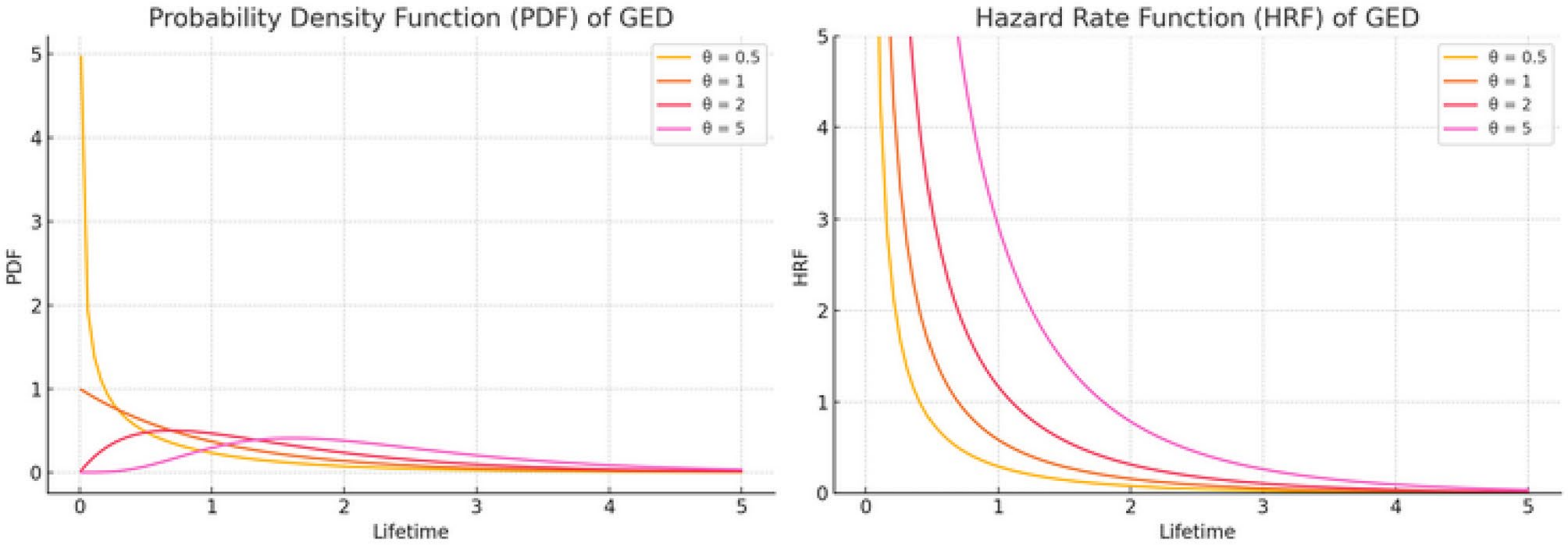
The PDF and HRF of GED for different values of the shape parameter $\theta$.

The GED is often considered a robust alternative to traditional distributions such as the Gamma and Weibull, particularly when modeling skewed data^32^. This distribution family boasts several advantageous properties, including:

1. Flexibility in modeling skewed lifetime data: The GED is well-suited for modeling lifetime data that exhibit right skewness, a common characteristic in reliability and survival studies (e.g., failure times of mechanical systems or biological survival times). Its concave density function and two parameters-scale ($\lambda$) and shape ($\theta$)-allow it to adapt to a variety of skewed distributions. The Weibull distribution also models skewed data effectively but involves more complex exponentiation in its CDF and PDF, which can complicate derivations in the OMELRRSS framework. The Gamma distribution is flexible but lacks a closed-form CDF, making it less tractable for exact inference under complex sampling schemes. GED’s simpler mathematical form facilitates closed-form solutions for likelihood functions and prediction intervals, which are essential for the computational demands of OMELRRSS.

2. Versatile hazard rate behavior: The GED’s hazard rate can be increasing, decreasing, or constant depending on the shape parameter $\theta$, offering significant flexibility to model diverse failure patterns. Figure 1 illustrates the PDF (left) and HRF (right) of the GED for different shape parameter values $\theta$. For PDF behavior: At $\theta = 0.5$, the distribution is right-skewed with higher initial probability. When $\theta = 1$, it follows an exponential decay. Higher $\theta$ values ($2, 5$) shift and spread the distribution, capturing diverse lifetime patterns. In addition, for HRF behavior: At $\theta = 0.5$, the failure rate decreases over time. When $\theta = 1$, it remains constant (exponential case). For $\theta > 1$, the HRF is increasing, meaning the failure rate grows over time. This confirms the GED’s flexibility in capturing various failure and survival patterns.

3. Computational efficiency in simulations: The GED allows for straightforward random number generation via the inverse CDF method, which is computationally efficient. This is particularly advantageous in our study, where extensive Monte Carlo simulations are used to assess the performance of estimators and prediction methods under OMELRRSS. In contrast: The Weibull distribution supports efficient simulation but involves additional complexity in handling its power terms. The Gamma distribution requires more intricate algorithms (e.g., rejection sampling), increasing computational overhead. GED’s efficiency in simulation supports the practical implementation of our methodology.

To clarify the distinctions between the sampling schemes, we define each method concisely and highlight its unique features: Classical RSS uses an auxiliary variable to rank units, selecting a subset for precise measurement to enhance estimation efficiency. MERSS modifies RSS by selecting the maximum or minimum from each ranked set, improving precision for extreme-value analysis. MELRRSS extends MERSS by incorporating lower $k$-record values, making it effective for extreme event analysis in lifetime data. OMELRRSS further refines MELRRSS by ordering samples to enable statistical inference, particularly under censoring, using order statistics. Classical RSS improves estimation efficiency through ranking. However, in reliability testing and extreme-event analysis, selecting extremes enhances precision. MERSS adapts RSS by focusing on extreme values, MELRRSS extends this by capturing lower $k$-record values, and ordered MELRRSS enables inference under censoring. This progression builds on RSS to address challenges in analyzing lifetime data with extreme events. We will introduce in detail the OMELRRSS method, a novel approach designed to enhance the efficiency of parameter estimation and prediction in the context of the GED. This method integrates the principles of RSS and MERSS, with the added complexity of incorporating lower $k$-record values and random sample sizes. OMELRRSS offers several advantages over traditional sampling methods, including improved efficiency in parameter estimation and reduced costs associated with data collection. By focusing on extreme values and incorporating random sample sizes, this method provides a robust framework for analyzing lifetime data in various applications. To obtain a MELRRSS of size $n$ under lower $k$-record values, follow these steps:

1. Initialization: Select fixed values for $k$, $n$, $a$, $m_1$, and $m_2$, ensuring that $m_1 + m_2 = n$.
2. Sampling: Draw two SRSs from the population, each of size $a$ (where $a > n$). The first sample consists of $m_1$ sequences, and the second sample consists of $m_2$ sequences.
3. Lower $k$-Record process: Given a sequence of iid RVs $\{X_1, X_2, X_3, \dots , X_a\}$, the lower $k$-record values are defined recursively: The first lower $k$-record occurs at the $k$th observation: $T_{1,k} = k,$ where $T_{1,k}$ represents the position of the first lower $k$-record. The corresponding lower $k$-record value is: $\omega _{(1)} = X_{T_{1,k}}.$ For $a \ge 1$, the position of the $(a+1)$th lower $k$-record is determined as: $T_{a+1,k} = \min \{ j > T_{a,k}: X_j < R_{a,k} \}.$ This means a new lower $k$-record occurs when an observation is smaller than the current record. The corresponding lower $k$-record value is: $\omega _{(a+1)} = X_{T_{a+1,k}}.$ The inter-$k$-record time, representing the number of observations needed to observe a new lower $k$-record, is given by: $\Delta T_{a,k} = T_{a+1,k} - T_{a,k}.$
4. Termination: The MELRRSS process consists of two phases: Phase 1: When the $i$th lower $k$-record is observed, terminate sampling for the $i$th sequence ($i = 1, \dots , m_1$). Phase 2: When the first lower $k$-record is observed, terminate sampling for the $j$th sequence ($j = 1, \dots , m_2$), where $m_2 \le m_1$.
5. Selection: Choose the $i$th lower $k$-record values from the first $m_1$ sequences ($i = 1, \dots , m_1$). Then, select the maximum lower $k$-record values from the last $m_2$ sequences.
6. Output: The resulting MELRRSS units are denoted by:
  $$\begin{aligned} \mathbf {\omega }_{MELRRSS} = \left( \omega _{(1)1}, \omega _{(2)2}, \dots , \omega _{(m_1)m_1}, \omega _{(1)1}, \omega _{(1)2}, \dots , \omega _{(1)m_2} \right) . \end{aligned}$$
  See Scheme 1 for a visual representation of this process.
  $$\begin{aligned}\begin{aligned} \underbrace{\left. \begin{array}{ccccccc} \underline{\omega _{(1)1}} & & & \\ \omega _{(1)2} & \underline{\omega _{(2)2}} & & \\ \vdots & \vdots & \ddots & \\ \omega _{(1)m_{1}} & \omega _{(2)m_{1}} & \ldots & \underline{\omega _{(m_{1})m_{1}}}\\ \underline{\omega _{(1)1}} & & & \\ \underline{\omega _{(1)2}} & & & \\ \vdots & & & \\ \underline{\omega _{(1)m_{2}}} & & & \\ \end{array}\right\} }_{\text {Judgment Rank}} \Rightarrow \underbrace{\left\{ \begin{array}{ccccccc} \omega _{(1)1} \\ \omega _{(2)2}\\ \vdots \\ \omega _{(m_{1})m_{1}}\\ \omega _{(1)1} \\ \omega _{(1)2}\\ \vdots \\ \omega _{(1)m_{2}}\\ \end{array}\right. }_{\text {MELRRSS}}\\ \mathrm {{\textbf {Scheme 1}}: An\ MELRRSS\ of \ sample\ size}\ n.\qquad \end{aligned} \end{aligned}$$
  The symbol $\omega _{(i) l}$ represents the $i$th lower $k$-record value from the $l$th sequence, where $k$ is a fixed positive integer determining the record level, $i = 1, \dots , m_1$, $j = 1, \dots , m_2$, and $l = i, j$. Note that these values are not necessarily ordered. The elements of the MELRRSS are INID RVs. This property arises due to the nature of the sampling process, where each sequence may produce distinct record values.
7. Ordered MELRRSS: The OMELRRSS is obtained by arranging the MELRRSS units in ascending order. Denote the ordered sample by: ${\textbf{z}} = \left( z_{1:n}, z_{2:n}, \dots , z_{n:n} \right) ,$ where $z_{1:n} \le z_{2:n} \le \dots \le z_{n:n}$. This ordering facilitates the application of statistical methods that rely on ordered data, such as estimation and prediction.
8. Incorporating randomness in sample sizes: To enhance flexibility in the data collection process, randomness is incorporated into the sample sizes. This approach is particularly useful in scenarios where practical constraints may lead to variability in the number of observations. The sample size $N$ is treated as a random variable, independent of the lifetimes of the units in the original sets^33^. The probability mass function (PMF) of $N$ is defined as: $$P(N = n) = \left\{ \begin{array}{ll} 1 - \frac{n - \rho }{\xi - \rho + 1}, & \text {if } n > \rho , \\ 1, & \text {if } n \le \rho , \end{array}\right.$$ where $\rho$ and $\xi$ are parameters of the distribution. This formulation allows for a flexible and realistic representation of sample size variability in practical applications.

Suppose $\mathbf {\omega }_{MELRRSS}$ is a MELRRSS drawn from a population following the GED under lower $k$-record sampling. Let $\omega _{(i)l}$ denote the $i$th lower $k$-record in the $l$th sequence. The PDF, CDF, and survival function (SF) of $\omega _{(i)l}$, denoted by $f_{(i)l}(\omega )$, $F_{(i)l}(\omega )$, and ${\overline{F}}_{(i)l}(\omega )$, respectively, correspond to the PDF, CDF, and SF of the $i$th lower $k$-records. These functions are given by (see^26^):

2.1 $$\begin{aligned} f_{(i)l}(\omega )=\frac{k^{i}}{(i-1)!}\left( -\log G(\omega ) \right) ^{i-1} (G(\omega ))^{k-1}g(\omega ),\quad 0<\omega <\infty , \end{aligned}$$

where: $g(\omega )$ and $G(\omega )$ are the PDF and CDF of the GED, respectively,

2.2 $$\begin{aligned} F_{(i)l}(\omega )=G(\omega )^{k}\sum ^{\infty }_{d=i}\frac{\left( -k\log G(\omega ) \right) ^{d}}{d!},\quad 0<\omega <\infty , \end{aligned}$$

and

2.3 $$\begin{aligned} \overline{F}_{(i)l}(\omega )=G(\omega )^{k}\sum ^{i-1}_{v=0}\frac{\left( -k\log G(\omega ) \right) ^{v}}{v!},\quad 0<\omega <\infty . \end{aligned}$$

The double type-II censored OMELRRSS can be constructed as follows: After arranging the OMELRRSS in ascending order, denoted by $z_{1:n} \le z_{2:n} \le \cdots \le z_{n:n}$, the smallest $r$ observations are censored below $z_{r}$, and the largest $s$ observations are censored above $z_{s}$. Only the middle $s - r + 1$ observations, $z_{r} \le z_{r+1} \le \cdots \le z_{s}$, are observed, where $1< r< s < n$. The resulting dataset is referred to as the double type-II censored OMELRRSS and is denoted by ${\textbf{z}} = (z_{r}, z_{r+1}, \ldots , z_{s})$. Following an idea introduced by Balakrishnan^34^ for order statistics derived from INID RVs, the joint density function (JDF) for OMELRRSS under double type-II censoring is given by:

2.4 $$\begin{aligned} \zeta (\theta ,\lambda |{{\textbf {z}}})=\frac{1}{(r-1)!(n-s)!}\mathbf {\sum _{p}}\left( \prod _{\kappa =1}^{r-1}F_{(i_{\kappa })j_{\kappa }}(z_{r})\prod _{\kappa =r}^{s}f_{(i_{\kappa })j_{\kappa }}(z_{\kappa })\prod _{\kappa =s+1}^{n}\left[ \overline{F}_{(i_{\kappa })j_{\kappa }}(z_{s})\right] \right) , \end{aligned}$$

where $\mathbf {\sum _{p}}$ accounts for all possible permutations $n!=(m_{1}+m_{2})!$ permutations $(i_{1}, i_{2},\ldots , i_{n})$ of the indices $(1, 2, \ldots , n)$, ensuring that the JDF captures the contributions of all possible orderings of the observed data. We can easily express the JDF as follows:

2.5 $$\begin{aligned} \zeta (\theta ,\lambda |{{\textbf {z}}})=\frac{1}{(r-1)!(n-s)!}\;\textrm{Per} (\mathbf {\nu }_{r,s,n}), \end{aligned}$$

where $\textrm{Per}(\mathbf {\nu }_{r,s,n}) = \sum _{p} \prod _{j=1}^{n} c_{j,i_{j}}$ represents the permanent of the real matrix $\mathbf {\nu }_{r,s,n} = (c_{i,j})$ of size $n \times n$,

2.6 $$\begin{aligned} \mathbf {\nu }_{r,s,n}=\left( \begin{array}{llllll} F_{(1)1}(z_{r})& \ldots & \quad F_{(m_{1})m_{1}}(z_{r})& \quad F_{(1)1}(z_{r})& \ldots & \quad F_{(1)m_{2}}(z_{r})\\ f_{(1)1}(z_{r})& \ldots & \quad f_{(m_{1})m_{1}}(z_{r})& \quad f_{(1)1}(z_{r})& \ldots & \quad f_{(1)m_{2}}(z_{r})\\ \vdots & \ddots & \qquad \vdots & \qquad \vdots & \ddots & \qquad \vdots \\ f_{(1)1}(z_{s})& \ldots & \quad f_{(m_{1})m_{1}}(z_{s})& \quad f_{(1)1}(z_{s})& \ldots & \quad f_{(1)m_{2}}(z_{s})\\ \overline{F}_{(1)1}(z_{s})& \ldots & \quad \overline{F}_{(m_{1})m_{1}}(z_{s})& \quad \overline{F}_{(1)1}(z_{s})& \ldots & \quad \overline{F}_{(1)m_{2}}(z_{s})\\ \end{array}\right) \begin{array}{llllll} \} (r-1)\;\textrm{rows} \\ \\ \\ \\ \} (n-s) \;\textrm{rows}. \end{array} \end{aligned}$$

By substituting the PDF, CDF, and SF defined in (2.1), (2.2), and (2.3) into (2.4), we can express the JDF of ${{\textbf {z}}}$ as follows:

2.7 $$\begin{aligned} \zeta (\theta ,\lambda |{{\textbf {z}}})= & \frac{1}{(r-1)!(n-s)!}\mathbf {\sum _{p}}\left( \prod _{\kappa _{1}=1}^{r-1}G(z_{r})^{k}\sum ^{\infty }_{l_{\kappa _{1}}=i_{\kappa _{1}}}\frac{\left( -k\log G(z_{r}) \right) ^{l_{\kappa _{1}}}}{l_{\kappa _{1}}!} \right. \nonumber \\\times & \left. \prod _{\kappa _{1}=r}^{s}\frac{k^{i_{\kappa _{1}}}}{(i_{\kappa _{1}}-1)!}\left( -\log G(z_{r}) \right) ^{i_{\kappa _{1}}-1} (G(z_{r}))^{k-1}g(z_{r}) \right. \nonumber \\\times & \left. \prod _{\kappa _{1}=s+1}^{n}G(z_{s})^{k}\sum ^{i_{\kappa _{1}}-1}_{v_{\kappa _{1}}=0}\frac{\left( -k\log G(z_{s}) \right) ^{v_{\kappa _{1}}}}{v_{\kappa _{1}}!}\right) . \end{aligned}$$

By utilizing the following identities:

$$\begin{aligned} \prod _{\kappa _{1}=1}^{r-1}\sum ^{\infty }_{l_{\kappa _{1}}=i_{\kappa _{1}}}\Upsilon _{l_{\kappa _{1}}}(i_{\kappa _{1}})=\sum ^{\infty }_{l_{1}=i_{1}}\sum ^{\infty }_{l_{2}=i_{2}}\cdots \sum ^{\infty }_{l_{r-1}=i_{r-1}}\prod _{\kappa _{1}=1}^{r-1}\Upsilon _{l_{\kappa _{1}}}(i_{\kappa _{1}}), \end{aligned}$$

and

$$\begin{aligned} \prod _{\kappa _{1}=s+1}^{n}\sum _{\upsilon _{\kappa _{1}}=0}^{i_{\kappa _{1}-1}}\varDelta _{\upsilon _{\kappa _{1}}}(i_{\kappa _{1}})=\sum _{\upsilon _{s+1}=0}^{i_{s+1}-1}\sum _{\upsilon _{s+2}=0}^{i_{s+2}-1}\cdots \sum _{\upsilon _{n}=0}^{i_{n}-1}\prod _{\kappa _{1}=s+1}^{n}\varDelta _{\upsilon _{\kappa _{1}}}(i_{\kappa _{1}}), \end{aligned}$$

where $i_{1}<i_{2}<\cdots <i_{n}$ are positive integers, the JDF in Equation (2.7) for the GED with parameters $\theta$ and $\lambda$, based on OMELRRSS with a random sample size, can be simplified as follows:

2.8 $$\begin{aligned} \zeta (\theta ,\lambda |{{\textbf {z}}})=\frac{1}{(r-1)!}\sum _{n=s}^{\infty }\mathbf {\sum _{p}}\sum _{{\underline{l}},\underline{\nu }}^{r,s,n} D_{{\underline{l}},\underline{\nu }}(r,s,n) V_{{\underline{l}},\underline{\nu }}(\lambda |{{\textbf {z}}})\theta ^{\eta _{{\underline{l}},\underline{\nu }}(r,s,n)} \exp \left( -\theta W_{{\underline{l}},\underline{\nu }}(\lambda |{{\textbf {z}}})\right) P_{s}(n), \end{aligned}$$

where, $$\displaystyle {\sum _{{\underline{l}},\underline{\nu }}^{r,s,n}=\sum ^{\infty }_{l_{1}=i_{1}}\sum ^{\infty }_{l_{2}=i_{2}}\cdots \sum ^{\infty }_{l_{r-1}=i_{r-1}}.\sum _{\upsilon _{s+1}=0}^{i_{s+1}-1}\sum _{\upsilon _{s+2}=0}^{i_{s+2}-1}\cdots \sum _{\upsilon _{n}=0}^{i_{n}-1}}$$, $${\underline{l}}=(l_{1},\ldots ,l_{r-1})$$, $$\underline{\nu }=(\nu _{s+1},\ldots ,\nu _{n})$$,

2.9 $$\begin{aligned} \eta _{{\underline{l}},\underline{\nu }}(r,s,n)= & \sum _{\kappa _{1}=1}^{r-1}l_{\kappa _{1}}+\sum _{\kappa _{1}=r}^{s}i_{\kappa _{1}}+\sum _{\kappa _{1}=s+1}^{n}\nu _{\kappa _{1}},\end{aligned}$$

2.10 $$\begin{aligned} D_{{\underline{l}},\underline{\nu }}(r,s,n)= & \left( \prod _{\kappa _{1}=1}^{r-1}\frac{\left( -k_{\kappa _{1}} \right) ^{l_{\kappa _{1}}}}{l_{\kappa _{1}}!(n-s)!}\right) \left( \prod _{\kappa _{1}=r}^{s} \frac{(-1)^{i_{\kappa _{1}-1}k_{\kappa _{1}}^{i_{\kappa _{1}}}}}{(i_{\kappa _{1}}-1)!}\right) \left( \prod _{\kappa _{1}=s+1}^{n}\frac{\left( -k_{\kappa _{1}}\right) ^{v_{\kappa _{1}}}}{v_{\kappa _{1}}!}\right) ,\end{aligned}$$

2.11 $$\begin{aligned} V_{{\underline{l}},\underline{\nu }}(\lambda |{{\textbf {z}}})= & \left( \log \left( 1-e^{-\lambda z_{r}} \right) ^{\sum _{\kappa _{1}=1}^{r-1}l_{\kappa _{1}}}\right) \left( \log \left( 1-e^{-\lambda z_{s}} \right) ^{\sum _{\kappa _{1}=s+1}^{n}\nu _{\kappa _{1}}}\right) \nonumber \\\times & \left( \prod _{\kappa _{1}=r}^{s}\frac{\lambda e^{-\lambda z_{\kappa _{1}}}\left( \log \left( 1-e^{-\lambda z_{\kappa _{1}}} \right) ^{i_{\kappa _{1}}-1}\right) }{\left( 1-e^{-\lambda z_{\kappa _{1}}} \right) } \right) ,\end{aligned}$$

2.12 $$\begin{aligned} W_{{\underline{l}},\underline{\nu }}(\lambda |{{\textbf {z}}})= & \sum _{\kappa _{1}=1}^{r-1}(-k_{\kappa _{1}})\log \left( 1-e^{-\lambda z_{r}} \right) +\sum _{\kappa _{1}=r}^{s}(-k_{\kappa _{1}})\log \left( 1-e^{-\lambda z_{\kappa _{1}}}\right) \nonumber \\+ & \sum _{\kappa _{1}=s+1}^{n}(-k_{\kappa _{1}})\log \left( 1-e^{-\lambda z_{s}} \right) . \end{aligned}$$

### Remark 1

It is important to note that the left-truncated distribution of $n$ at $s$ is represented by $P_{s}(n) = P(N = n \mid N \ge s)$ in Eq. (2.8). We assume that $N$ follows a discrete uniform distribution with parameters $\rho$ and $\xi$, denoted as $N \sim \textrm{Uniform}(\rho , \xi )$, where $N$ is a positive integer-valued random variable^33^. Consequently, the conditional probability $P_{s}(n)$ can be expressed as $P_{s}(n) = ((\xi - \rho + 1)P(N \ge s))^{-1},$ where $P(N \ge s)$ is given by:

$$\begin{aligned} P(N \ge s) = \left\{ \begin{array}{ll} 1, & \textrm{if }\; s \le \rho , \\ \\ 1 - \frac{s - \rho }{\xi - \rho + 1}, & \textrm{if }\; s > \rho . \end{array} \right. \end{aligned}$$

## Maximum likelihood estimation

To obtain the MLE of the unknown parameter $\theta$, assuming the scale parameter $\lambda$ is known, we begin by applying the natural logarithm ($\log$) to the likelihood function given in Eq. (2.8). This yields the log-likelihood function:

3.1 $$\begin{aligned} \log \zeta (\theta |{{\textbf {z}}}) \propto \log \left( \sum _{n=s}^{\infty } \mathbf {\sum _{p}} \sum _{{\underline{l}},\underline{\nu }}^{r,s,n} D_{{\underline{l}},\underline{\nu }}(r,s,n) V_{{\underline{l}},\underline{\nu }}(\lambda |{{\textbf {z}}}) \, \theta ^{\eta _{{\underline{l}},\underline{\nu }}(r,s,n)} \exp \left( -\theta W_{{\underline{l}},\underline{\nu }}(\lambda |{{\textbf {z}}})\right) P_{s}(n) \right) . \end{aligned}$$

The log-likelihood function combines contributions from observed data (via PDF) and censored data (via CDF), summing over all possible sample permutations. The MLE of $\theta$, denoted as ${\tilde{\theta }}_{MLE}$, is obtained by differentiating the log-likelihood function in Eq. (3.1) with respect to $\theta$, setting the derivative equal to zero, and solving for $\theta$. Specifically, ${\tilde{\theta }}_{MLE}$ is the value of $\theta$ that maximizes the expression in Eq. (3.1). However, the derivative of Eq. (3.1) with respect to $\theta$ results in a nonlinear equation for the unknown parameter $\theta$. Due to the complexity of the equation, it is evident that a closed-form solution for ${\tilde{\theta }}_{MLE}$ is difficult to obtain analytically. Therefore, a numerical approach is required to solve the equation. To address this, we employ an iterative numerical procedure, such as the Newton-Raphson method, to approximate the MLE of $\theta$. This method is particularly suitable for solving nonlinear equations and can efficiently converge to the desired solution when provided with appropriate initial values. The iterative procedure is applied for given values of $(r, s, n, \lambda , {{\textbf {z}}}, \rho )$, ensuring that the estimated ${\tilde{\theta }}_{MLE}$ maximizes the log-likelihood function in Eq. (3.1).

## Empirical Bayes estimation

We explore Bayesian estimation methods under the assumption that the random variable $\Theta$ (with realizations $\theta$) follows an exponential prior distribution. This prior distribution is represented as:

4.1 $$\begin{aligned} \hbar (\theta ) = b \exp \left( -b\theta \right) , \quad \theta> 0, \; (b > 0), \end{aligned}$$

where $b$ is a hyper-parameter chosen to incorporate prior knowledge about $\theta$. The exponential prior is particularly advantageous due to its flexibility and simplicity, making it suitable for capturing a wide range of the experimenter’s prior beliefs. For further discussion on the use of exponential priors, see^35–38^. Using the likelihood function from Eq. (2.8) and the prior density from Eq. (4.1), the posterior density of $\theta$ can be derived as:

4.2 $$\begin{aligned} \Re (\theta |{{\textbf {z}}}) = \frac{1}{\Bbbk _{{\underline{l}},\underline{\nu }}(r,s)} \sum _{n=s}^{\infty } \mathbf {\sum _{p}} \sum _{{\underline{l}},\underline{\nu }}^{r,s,n} D_{{\underline{l}},\underline{\nu }}(r,s,n) V_{{\underline{l}},\underline{\nu }}(\lambda |{{\textbf {z}}}) \, \theta ^{\eta _{{\underline{l}},\underline{\nu }}(r,s,n)} \exp \left[ -\left( b + W_{{\underline{l}},\underline{\nu }}(\lambda |{{\textbf {z}}})\right) \theta \right] P_{s}(n), \end{aligned}$$

where $\Bbbk _{{\underline{l}},\underline{\nu }}(r,s)$ is the normalizing constant ensuring that the posterior density integrates to one. The normalizing constant is given by:

$$\begin{aligned} \Bbbk _{{\underline{l}},\underline{\nu }}(r,s)= & \sum _{n=s}^{\infty } \mathbf {\sum _{p}} \sum _{{\underline{l}},\underline{\nu }}^{r,s,n} D_{{\underline{l}},\underline{\nu }}(r,s,n) V_{{\underline{l}},\underline{\nu }}(\lambda |{{\textbf {z}}}) \left( b + W_{{\underline{l}},\underline{\nu }}(\lambda |{{\textbf {z}}})\right) ^{-(\eta _{{\underline{l}},\underline{\nu }}(r,s,n)+1)} \nonumber \\\times & \Gamma \left( \eta _{{\underline{l}},\underline{\nu }}(r,s,n)+1\right) P_{s}(n), \end{aligned}$$

where $\Gamma (\cdot )$ denotes the gamma function. The posterior density $\Re (\theta |{{\textbf {z}}})$ combines the prior information with the observed data ${{\textbf {z}}}$ to provide an updated distribution for $\theta$, reflecting both prior beliefs and empirical evidence. In Bayesian analysis, the choice of loss function is critical as it directly influences the estimation process. To comprehensively compare Bayesian estimates, we employ two distinct types of loss functions: the squared error (SE) loss function and the LINEX loss function. These loss functions for the model parameter $\Theta$ are defined as follows:

$$\begin{aligned} \ell _{SR}(\Theta , {\tilde{\Theta }})= & \left( \Theta - {\tilde{\Theta }}\right) ^{2}, \\ \ell _{LINEX}(\Theta , {\tilde{\Theta }})= & e^{c({\tilde{\Theta }} - \Theta )} - c({\tilde{\Theta }} - \Theta ) - 1, \quad c \ne 0, \end{aligned}$$

where ${\tilde{\Theta }}$ represents an estimate of $\Theta$. The parameter $c$ in the LINEX loss function controls the degree of asymmetry. For $c > 0$, the LINEX loss function is asymmetric around zero, penalizing overestimation more severely than underestimation. Conversely, for $c < 0$, the opposite holds true. When $c$ is close to zero, the LINEX loss function approximates the symmetric SE loss function, yielding similar estimates. Due to its flexibility in handling asymmetry, the LINEX loss function is particularly well-suited for modeling lifespan data. As highlighted in^39,40^, overestimation of survival and failure rate functions is often more critical than underestimation in reliability and survival analysis. The Bayes estimate of a function $T = T(\Theta )$ under the SE and LINEX loss functions is given by:

$$\begin{aligned} {\tilde{T}}_{SE}= & E(T(\Theta )|{{\textbf {z}}}) = \int _{\Theta } T(\Theta ) \, \Re (\Theta |{{\textbf {z}}}) \, d\Theta , \\ {\tilde{T}}_{LINEX}= & \frac{-1}{c} \log \left( E(e^{-cT(\Theta )} |{{\textbf {z}}})\right) = \frac{-1}{c} \log \left( \int _{\Theta } e^{-cT(\Theta )} \, \Re (\Theta |{{\textbf {z}}}) \, d\Theta \right) , \end{aligned}$$

where $\Re (\Theta |{{\textbf {z}}})$ is the posterior density of $\Theta$ given the data ${{\textbf {z}}}$. Using these results, the Bayes estimators of the unknown parameter $\theta$ under the SE and LINEX loss functions are derived as:

4.3 $$\begin{aligned} {\tilde{\theta }}_{SE}= & \frac{1}{\Bbbk _{{\underline{l}},\underline{\nu }}(r,s)} \sum _{n=s}^{\infty } \mathbf {\sum _{p}} \sum _{{\underline{l}},\underline{\nu }}^{r,s,n} D_{{\underline{l}},\underline{\nu }}(r,s,n) V_{{\underline{l}},\underline{\nu }}(\lambda |{{\textbf {z}}}) \left( b + W_{{\underline{l}},\underline{\nu }}(\lambda |{{\textbf {z}}})\right) ^{-(\eta _{{\underline{l}},\underline{\nu }}(r,s,n)+2)} \nonumber \\\times & \Gamma \left( \eta _{{\underline{l}},\underline{\nu }}(r,s,n)+2\right) P_{s}(n), \end{aligned}$$

4.4 $$\begin{aligned} {\tilde{\theta }}_{LINEX}= & \frac{-1}{c} \log \left( \frac{1}{\Bbbk _{{\underline{l}},\underline{\nu }}(r,s)} \sum _{n=s}^{\infty } \mathbf {\sum _{p}} \sum _{{\underline{l}},\underline{\nu }}^{r,s,n} D_{{\underline{l}},\underline{\nu }}(r,s,n) V_{{\underline{l}},\underline{\nu }}(\lambda |{{\textbf {z}}}) \left( b + c + W_{{\underline{l}},\underline{\nu }}(\lambda |{{\textbf {z}}})\right) ^{-(\eta _{{\underline{l}},\underline{\nu }}(r,s,n)+1)} \right. \nonumber \\\times & \left. \Gamma \left( \eta _{{\underline{l}},\underline{\nu }}(r,s,n)+1\right) P_{s}(n)\right) . \end{aligned}$$

Here, $\Bbbk _{{\underline{l}},\underline{\nu }}(r,s)$ is the normalizing constant defined earlier, and $\Gamma (\cdot )$ denotes the gamma function. These estimators provide a way to incorporate the posterior distribution and the chosen loss function to obtain optimal estimates of $\theta$.

Unlike traditional Bayesian methods that require fully specified priors, empirical Bayes estimation uses the data to estimate prior parameters, making it ideal when prior knowledge is limited. In this study, we adopt the balanced loss function, initially introduced by Zellner^38^. The balanced loss function is defined as:

$$\begin{aligned} L^{*}(\Theta , \omega ) = \Delta \varrho (\omega _{o}, \omega ) + (1 - \Delta ) \varrho (\Theta , \omega ), \end{aligned}$$

where: $\varrho (\cdot )$ is an arbitrary loss function, $\omega _{o}$ is a chosen estimate of $\omega$, and $\Delta$ is a weight such that $0 \le \Delta \le 1$. The balanced loss function combines two components: one that measures the deviation of the estimate $\omega$ from a target estimate $\omega _{o}$, and another that measures the deviation of $\omega$ from the true parameter $\Theta$. This formulation allows for a flexible balance between these two objectives. When $\varrho (\Theta , \omega ) = (\Theta - \omega )^{2}$, the balanced loss function simplifies to the balanced squared error (BSE) loss function. The corresponding Bayes estimator under the BSE loss function is given by:

$$\begin{aligned} {\tilde{T}}_{BSE} = \Delta {\tilde{T}} + (1 - \Delta ) E(T(\Theta )|{{\textbf {z}}}), \end{aligned}$$

where ${\tilde{T}}$ represents the MLE of $T$, and $E(T(\Theta )|{{\textbf {z}}})$ is the posterior expectation of $T(\Theta )$ given the data ${{\textbf {z}}}$. Similarly, when $\varrho (\Theta , \omega ) = e^{c(\Theta - \omega )} - c(\Theta - \omega ) - 1$, the balanced loss function reduces to the balanced LINEX (BLINEX) loss function, defined as:

$$\begin{aligned} L^{*}(\Theta , \omega ) = \Delta \left( e^{c(\Theta - \omega )} - c(\Theta - \omega ) - 1\right) + (1 - \Delta ) \left( e^{c(\Theta - \omega )} - c(\Theta - \omega ) - 1\right) , \quad c \ne 0, \end{aligned}$$

where $c$ is the shape parameter controlling the asymmetry of the BLINEX loss function. Using these loss functions, the Bayes estimators of the unknown parameter $\theta$ under the BSE and BLINEX loss functions are derived as:

4.5 $$\begin{aligned} {\tilde{\theta }}_{BSE}= & \Delta {\tilde{\theta }}_{MLE} + \frac{1 - \Delta }{\Bbbk _{{\underline{l}},\underline{\nu }}(r,s)} \sum _{n=s}^{\infty } \mathbf {\sum _{p}} \sum _{{\underline{l}},\underline{\nu }}^{r,s,n} D_{{\underline{l}},\underline{\nu }}(r,s,n) V_{{\underline{l}},\underline{\nu }}(\lambda |{{\textbf {z}}}) \nonumber \\\times & \left( b + W_{{\underline{l}},\underline{\nu }}(\lambda |{{\textbf {z}}})\right) ^{-(\eta _{{\underline{l}},\underline{\nu }}(r,s,n)+2)} \Gamma \left( \eta _{{\underline{l}},\underline{\nu }}(r,s,n)+2\right) P_{s}(n), \end{aligned}$$

4.6 $$\begin{aligned} {\tilde{\theta }}_{BLINEX}= & \frac{-1}{c} \log \left( \Delta e^{-c{\tilde{\theta }}_{MLE}} + \frac{1 - \Delta }{\Bbbk _{{\underline{l}},\underline{\nu }}(r,s)} \sum _{n=s}^{\infty } \mathbf {\sum _{p}} \sum _{{\underline{l}},\underline{\nu }}^{r,s,n} D_{{\underline{l}},\underline{\nu }}(r,s,n) V_{{\underline{l}},\underline{\nu }}(\lambda |{{\textbf {z}}}) \right. \nonumber \\\times & \left. \left( b + c + W_{{\underline{l}},\underline{\nu }}(\lambda |{{\textbf {z}}})\right) ^{-(\eta _{{\underline{l}},\underline{\nu }}(r,s,n)+1)} \Gamma \left( \eta _{{\underline{l}},\underline{\nu }}(r,s,n)+1\right) P_{s}(n) \right) . \end{aligned}$$

In Eqs. (4.5) and (4.6), the hyper-parameter $b$ is an unknown parameter, which prevents the direct use of $\theta$ in the estimation process. To address this, we employ the MLE approach to estimate the value of $b$. This method has been widely used to estimate hyperparameters of prior distributions; for further details, see^41^. Multiply the PDF of the GED by the prior distribution given in Eq. (4.1) and then integrate over $\theta$; we can obtain the marginal PDF and CDF of $b$. Hence, substituting the marginal PDF and CDF of $b$ into Eq. (2.7) yields the likelihood function for $b$. This allows us to apply the MLE approach to estimate the hyper-parameter ${\tilde{b}}$ of the prior distribution. Once ${\tilde{b}}$ is estimated, the Bayes estimators of the unknown parameter $\theta$ under the BSE and BLINEX loss functions are given by:

4.7 $$\begin{aligned} {\tilde{\theta }}_{BSE}= & \Delta {\tilde{\theta }}_{MLE} + \frac{1 - \Delta }{\Bbbk _{{\underline{l}},\underline{\nu }}(r,s)} \sum _{n=s}^{\infty } \mathbf {\sum _{p}} \sum _{{\underline{l}},\underline{\nu }}^{r,s,n} D_{{\underline{l}},\underline{\nu }}(r,s,n) V_{{\underline{l}},\underline{\nu }}(\lambda |{{\textbf {z}}}) \nonumber \\\times & \left( {\tilde{b}} + W_{{\underline{l}},\underline{\nu }}(\lambda |{{\textbf {z}}})\right) ^{-(\eta _{{\underline{l}},\underline{\nu }}(r,s,n)+2)} \Gamma \left( \eta _{{\underline{l}},\underline{\nu }}(r,s,n)+2\right) P_{s}(n), \end{aligned}$$

4.8 $$\begin{aligned} {\tilde{\theta }}_{BLINEX}= & \frac{-1}{c} \log \left( \Delta e^{-c{\tilde{\theta }}_{MLE}} + \frac{1 - \Delta }{\Bbbk _{{\underline{l}},\underline{\nu }}(r,s)} \sum _{n=s}^{\infty } \mathbf {\sum _{p}} \sum _{{\underline{l}},\underline{\nu }}^{r,s,n} D_{{\underline{l}},\underline{\nu }}(r,s,n) V_{{\underline{l}},\underline{\nu }}(\lambda |{{\textbf {z}}}) \right. \nonumber \\\times & \left. \left( {\tilde{b}} + c + W_{{\underline{l}},\underline{\nu }}(\lambda |{{\textbf {z}}})\right) ^{-(\eta _{{\underline{l}},\underline{\nu }}(r,s,n)+1)} \Gamma \left( \eta _{{\underline{l}},\underline{\nu }}(r,s,n)+1\right) P_{s}(n) \right) , \end{aligned}$$

where: ${\tilde{b}}$ is the estimated hyper-parameter obtained using the MLE approach.

## Exact prediction intervals based on lower OMELRRSS

In this section, we employ the pivotal approach to construct prediction intervals for the unknown future lower OMELRRSS values $z_{\tau :n}$, where $\tau = s+1, s+2, \ldots , n$. These intervals are based on the observed values $z_{i:n}$, where $i = r, r+1, \ldots , s$. The pivotal approach relies on a pivotal quantity, which is an explicit function of the observed lower values $z_{r:n} \ge z_{r+1:n} \ge \cdots \ge z_{s:n}$ and the unknown future OMELRRSS $z_{\tau :n}$. Importantly, the pivotal function must be invertible and have a well-defined CDF. For further details, see Barakat and Newer^31^. Pivotal quantities are essential tools for developing prediction intervals for future lower OMELRRSS. If $A$ is a pivotal quantity for the future lower OMELRRSS $z_{\tau :n}$, then for any $0< \pi < 1$, there exist constants $a_1$ and $a_2$ (depending on $\pi$) and two functions $\ell _1(z_{r:n}, z_{r+1:n}, \ldots , z_{s:n})$ and $\ell _2(z_{r:n}, z_{r+1:n}, \ldots , z_{s:n})$ (independent of $z_{\tau :n}$) such that:

$$\begin{aligned} 1 - P\left( \ell _1(z_{r:n}, z_{r+1:n}, \ldots , z_{s:n})< z_{\tau :n} \le \ell _2(z_{r:n}, z_{r+1:n}, \ldots , z_{s:n})\right) = 1 - P(a_1 < A \le a_2) = \pi . \end{aligned}$$

The interval $[\ell _1, \ell _2]$ constitutes a $(1-\pi ) \times 100\%$ predictive confidence interval (PCI) for $z_{\tau :n}$. We propose the following pivotal quantity based on $\text{ GED }(\theta , \lambda )$:

5.1 $$\begin{aligned} \Psi ^{OMELRRSS}_{s,\tau :n} = \frac{\log \left( 1 - e^{-\lambda z_{\tau :n}}\right) - \log \left( 1 - e^{-\lambda z_{s:n}}\right) }{\log \left( 1 - e^{-\lambda z_{s:n}}\right) }, \quad 1 \le s < \tau \le n. \end{aligned}$$

### Remark 2

The pivotal quantities defined in Eq. (5.1) are scale-free and shape-free. This property allows us to apply these pivotal quantities to any $\text{ GED }(\theta , \lambda )$ distribution, regardless of the unknown scale parameter $\lambda$ or the unknown shape parameter $\theta$.

### The distribution function of the pivotal statistic based on lower OMELRRSS

In this subsection, we derive the PDF and CDF of the pivotal statistic $\Psi ^{OMELRRSS}_{s,\tau :n}$ based on the $\text{ GED }(\theta ,\lambda )$. We assume that the sample size is a positive integer-valued RV that is independent of the lifetimes of the $n$ units in the original $n$ SRSs. Let $z_{i:n}$, $i = 1, 2, \ldots , n$, represent the OMELRRSS values. These are obtained by arranging the values $\omega _{i(i:n)}$, $i = 1, 2, \ldots , n$, in descending order of magnitude. Using results for lower order statistics from INID RVs (see Balakrishnan^34^), the JDF of $z_{s:n}$ and $z_{\tau :n}$, where $1 \le s < \tau \le n$, is given by:

5.2 $$\begin{aligned} f_{s,\tau :n}^\star (x, y) = \frac{1}{(s-1)!(\tau -s-1)!(n-\tau )!} \, \textrm{Per} \, {\textbf{A}}_{s,\tau ,n}, \quad x > y, \end{aligned}$$

where: $\textrm{Per} \, {\textbf{A}}_{s,\tau ,n}$ denotes the permanent of the matrix ${\textbf{A}}_{s,\tau ,n}$, which is constructed based on the INID RVs, $x$ and $y$ are the observed values of $z_{s:n}$ and $z_{\tau :n}$, respectively,

$$\begin{aligned} {\textbf{A}}_{s,\tau ,n}=\left( \begin{array}{llllll} \overline{F}_{1:1}(x)& \ldots & \quad \overline{F}_{m_{1}:m_{1}}(x)& \quad \overline{F}_{1:1}(x)& \ldots & \quad \overline{F}_{1:m_{2}}(x)\\ f_{1:1}(x)& \ldots & \quad f_{m_{1}:m_{1}}(x)& \quad f_{1:1}(x)& \ldots & \quad f_{1:m_{2}}(x)\\ F^\star _{1:1}(x)& \ldots & \quad F^\star _{m_{1}:m_{1}}(x)& \quad F^\star _{1:1}(x)& \ldots & \quad F^\star _{1:m_{2}}(x)\\ f_{1:1}(y)& \ldots & \quad f_{m_{1}:m_{1}}(y)& \quad f_{1:1}(y)& \ldots & \quad f_{1:m_{2}}(y)\\ F_{1:1}(y)& \ldots & \quad F_{m_{1}:m_{1}}(y)& \quad F_{1:1}(y)& \ldots & \quad F_{1:m_{2}}(y)\\ \end{array}\right) \begin{array}{lllll} \\ \}(s-1)\;\textrm{rows} \\ \\ \}(\tau -s-1)\;\textrm{rows} \\ \\ \}(n-\tau )\;\textrm{rows}, \\ \\ \end{array} \end{aligned}$$

$F^\star _{i:i}(x)=F_{i:i}(x)-F_{i:i}(y)$. Therefore, we can easily express the JDF of $z_{s:n}$ and $z_{\tau :n}$, $1\le s<\tau \le n$, as

5.3 $$\begin{aligned} f_{s,\tau :n}^\star (x,y)= & \frac{1}{(s-1)!(\tau -s-1)!(n-\tau )!}\mathbf {\sum _{P}} \left( \left[ \prod _{\iota =1}^{s-1} \overline{F}_{i_{\iota }:i_{\iota }}(x)\right] f_{i_{s}:i_{s}}(x)\right. \nonumber \\\times & \left. \left[ \prod _{\iota =s+1}^{\tau -1}\left[ F_{i_{\iota }:i_{\iota }}(x)- F_{i_{\iota }:i_{\iota }}(y)\right] \right] f_{i_{\tau }:i_{\tau }}(y)\prod _{\iota =\tau +1}^{n}F_{i_{\iota }:i_{\iota }}(y)\right) \textrm{I}(y<x), \end{aligned}$$

where $\textrm{I}(y<x)$ is the usual indicator function. Substituting the PDF, CDF, and SF defined in Eqs. (2.1), (2.2), and (2.3), respectively, into Eq. (5.3), the JDF of $z_{s:n}$ and $z_{\tau :n}$, where $1 \le s < \tau \le n$, can be expressed as:

5.4 $$\begin{aligned} f_{s,\tau :n}^\star (x,y)= & \frac{1}{(s-1)!(\tau -s-1)!(n-\tau )!}\mathbf {\sum _{P}} \left( \left[ \prod _{\iota =1}^{s-1}G(x)^{k_{\iota }}\sum ^{i_{\iota }-1}_{l_{\iota }=0}\frac{\left( -k_{\iota }\log G(x) \right) ^{l_{\iota }}}{l_{\iota }!}\right] \right. \nonumber \\\times & \left. \frac{k^{i_{s}}}{(i_{s}-1)!}\left( -\log G(x) \right) ^{i_{s}-1}(G(x))^{k-1}g(x) \right. \nonumber \\\times & \left. \left[ \prod _{\iota =s+1}^{\tau -1}\sum ^{\infty }_{v_{\iota }=i_{\iota }}\frac{\left( -k_{\iota } \right) ^{v_{\iota }}}{v_{\iota }!}\left( G(x)^{k_{\iota }}(\log G(x))^{v_{\iota }}- G(y)^{k_{\iota }}(\log G(y))^{v_{\iota }}\right) \right] \right. \nonumber \\\times & \left. \frac{k^{i_{\tau }}}{(i_{\tau }-1)!}\left( -\log G(y) \right) ^{i_{\tau }-1}(G(y))^{k-1}g(y) \right. \nonumber \\\times & \left. \left[ \prod _{\iota =\tau +1}^{n}G(y)^{k_{\iota }}\sum ^{\infty }_{\nu _{\iota }=i_{\iota }}\frac{\left( -k_{\iota }\log G(y) \right) ^{\nu _{\iota }}}{\nu _{\iota }!}\right] \right) \textrm{I}(y<x). \end{aligned}$$

Thus, the JDF in Eq. (5.4), based on the $\text{ GED }(\theta , \lambda )$, can be simplified as,

5.5 $$\begin{aligned} f_{s,\tau :n}^\star (x,y)= & \frac{\lambda ^{2}e^{-\lambda (x+y)}}{(s-1)!(\tau -s-1)!(n-\tau )!}\mathbf {\sum _{P}}\sum _{{\underline{l}},{\underline{i}},\underline{\textrm{v}},{\underline{h}},\underline{\nu }}^{s,\tau ,n}\textrm{Q}_{{\underline{l}},{\underline{i}},\underline{\textrm{v}},{\underline{h}},\underline{\nu }}(s,\tau ,n) \nonumber \\\times & \theta ^{\sum _{\iota =1}^{s-1}l_{\iota }+\sum _{\iota =s+1}^{\tau -1}v_{\iota }+\sum _{\iota =\tau +1}^{n}\nu _{\iota }+i_{s}+i_{\tau }} \nonumber \\\times & \left( 1-e^{-\lambda x}\right) ^{\left( \sum _{\iota =1}^{s-1}k_{\iota }+\sum _{\iota =s+1}^{\tau -1}k_{\iota }(1-h_{\iota })+k \right) \theta -1} \nonumber \\\times & \left( \log \left( 1-e^{-\lambda x}\right) \right) ^{\sum _{\iota =1}^{s-1}l_{\iota }+\sum _{\iota =s+1}^{\tau -1}v_{\iota }(1-h_{\iota })+i_{r}-1} \nonumber \\\times & \left( 1-e^{-\lambda y}\right) ^{\left( \sum _{\iota =s+1}^{\tau -1}k_{\iota }h_{\iota }+\sum _{\iota =\tau +1}^{n}k_{\iota }+k \right) \theta -1} \nonumber \\\times & \left( \log \left( 1-e^{-\lambda y}\right) \right) ^{\sum _{\iota =s+1}^{\tau -1}h_{\iota }v_{\iota }+\sum _{\iota =\tau +1}^{n}\nu _{\iota }+i_{\tau }-1}\textrm{I}(y<x), \end{aligned}$$

where $${\underline{l}}=(l_{1},l_{2}, \ldots ,l_{s-1})$$, $${\underline{i}}=(i_{1},i_{2},\ldots ,i_{s-1}),$$ $${\underline{v}}=(v_{s+1},v_{s+2}, \ldots ,v_{\tau -1})$$,

$${\underline{h}}=(h_{s+1},h_{s+2},\ldots ,h_{\tau -1}),$$ $$\underline{\nu }=(\nu _{\tau +1},\nu _{\tau +2},\ldots ,\nu _{n}),$$

$$\begin{aligned} \sum _{{\underline{l}},{\underline{i}},\underline{\textrm{v}},{\underline{h}},\underline{\nu }}^{s,\tau ,n}= & \sum ^{i_{1}-1}_{l_{1}=0} \sum ^{i_{2}-1}_{l_{2}=0}\cdots \sum ^{i_{s-1}-1}_{l_{s-1}=0} \sum ^{\infty }_{v_{s+1}=i_{s+1}}\sum ^{\infty }_{v_{s+2}=i_{s+2}}\cdots \sum ^{\infty }_{v_{\tau -1}=i_{\tau -1}} \sum ^{1}_{h_{s+1}=0}\sum ^{1}_{h_{s+2}=0}\cdots \sum ^{1}_{h_{\tau -1}=0}\\\times & \sum ^{\infty }_{\nu _{\tau +1}=i_{\tau +1}}\sum ^{\infty }_{\nu _{\tau +2}=i_{\tau +2}}\cdots \sum ^{\infty }_{\nu _{n}=i_{n}}, \end{aligned}$$

$$\begin{aligned} \textrm{Q}_{{\underline{l}},{\underline{i}},\underline{\textrm{v}},{\underline{h}},\underline{\nu }}(s,\tau ,n)= & \frac{k^{i_{s}}k^{i_{\tau }}(-1)^{\sum _{\iota =s+1}^{\tau -1}h_{\iota }+i_{s}+i_{\tau }-2}}{(i_{s}-1)!(i_{\tau }-1)!} \left( \prod _{\iota =1}^{s-1} \frac{(-k_{\iota })^{l_{\iota }}}{l_{\iota }!}\right) \left( \prod _{\iota =s+1}^{\tau -1} \frac{(-k_{\iota })^{v_{\iota }}}{v_{\iota }!}\right) \nonumber \\\times & \left( \prod _{\iota =\tau +1}^{n} \frac{(-k_{\iota })^{\nu _{\iota }}}{\nu _{\iota }!}\right) . \end{aligned}$$

#### Theorem 5.1.1

*Suppose that* $z_{r:N}\ge z_{r+1:N}\ge \cdots \ge z_{s:N}$ *are the first observed lower OMELRRSS based on* $\text{ GED }(\theta ,\lambda )$, *where N is a positive integer-valued RV that is independent on all the lifetimes of the n SRSs. Then, the SF *$\mathrm {F_{\Psi ^{OMELRRSS}_{s,\tau :N}}}$ *of the pivotal quantity* $\Psi ^{OMELRRSS}_{s,\tau :N}$ *is given by*:

5.6 $$\begin{aligned} \mathrm {F_{\Psi ^{OMELRRSS}_{s,\tau :N}}}(x)= & 1-\left( (s-1)!(\tau -s-1)!\textrm{P}(N\ge \tau )\right) ^{-1} \sum ^{\infty }_{n=\tau }\mathbf {\sum _{P}}\sum _{{\underline{l}},{\underline{i}},\underline{\textrm{v}},{\underline{h}},\underline{\nu }}^{s,\tau ,n}\nonumber \\\times & \frac{\textrm{Q}^{\star }_{{\underline{l}},{\underline{i}},\underline{\textrm{v}},{\underline{h}},\underline{\nu }}(s,\tau ,n)\Gamma (1+V_{s,\tau ,n}(\theta ))}{D_{s,\tau ,n}(\theta )-T_{s,\tau ,n}(\theta )}\left[ \left( T_{s,\tau ,n}(\theta )\right) ^{1-W_{s,\tau ,n}(\theta )} \right. \nonumber \\\times & \left. \Upsilon \left( 1,2+V_{s,\tau ,n}(\theta )-W_{s,\tau ,n}(\theta ),2+V_{s,\tau ,n}(\theta ),\frac{D_{s,\tau ,n}(\theta )}{D_{s,\tau ,n}(\theta )-T_{s,\tau ,n}(\theta )}\right) \right. \nonumber \\- & \left. \left( 1+\Psi \right) ^{1+V_{s,\tau ,n}(\theta )}\left( T_{s,\tau ,n}(\theta )+D_{s,\tau ,n}(\theta )\Psi \right) ^{1-W_{s,\tau ,n}(\theta )} \right. \nonumber \\\times & \left. \Upsilon \left( 1,2+V_{s,\tau ,n}(\theta )-W_{s,\tau ,n}(\theta ),2+V_{s,\tau ,n}(\theta ),\frac{D_{s,\tau ,n}(\theta )\left( 1+\Psi \right) }{D_{s,\tau ,n}(\theta )-T_{s,\tau ,n}(\theta )}\right) \right] \nonumber \\\times & \textrm{P}(N=n),\; x \ge 0, \end{aligned}$$

*where* $\textrm{Q}^{\star }_{{\underline{l}},{\underline{i}},\underline{\textrm{v}},{\underline{h}},\underline{\nu }}(s,\tau ,n)=\textrm{Q}_{{\underline{l}},{\underline{i}},\underline{\textrm{v}},{\underline{h}},\underline{\nu }}(s,\tau ,n)\theta ^{W_{s,\tau ,n}(\theta )}\Gamma (W_{s,\tau ,n}(\theta ))((n-\tau )!)^{-1}$, $W_{s,\tau ,n}(\theta )=\sum _{\iota =1}^{s-1}l_{\iota }+\sum _{\iota =s+1}^{\tau -1}v_{\iota }+\sum _{\iota =\tau +1}^{n}\nu _{\iota }+i_{s}+i_{\tau }$, $D_{s,\tau ,n}(\theta )=-\theta \left( \sum _{\iota =s+1}^{\tau -1}k_{\iota }h_{\iota }+\sum _{\iota =\tau +1}^{n}k_{\iota }+k\right)$, $V_{s,\tau ,n}(\theta )=\sum _{\iota =s+1}^{\tau -1}h_{\iota }v_{\iota }+\sum _{\iota =\tau +1}^{n}\nu _{\iota }+i_{s}-1$, $T_{s,\tau ,n}(\theta )=-\theta \left( \sum _{\iota =1}^{s-1}k_{\iota }+\sum _{\iota =s+1}^{\tau -1}k_{\iota }+\sum _{\iota =\tau +1}^{n}k_{\iota }+2k \right)$, *and* $\Upsilon (.)$ *hypergeometric 2F1 regularized function*.

*Furthermore, the *$\mathrm {\overline{F}_{\Psi ^{OMELRRSS}_{s,\tau :N}}}(\psi )=1-\pi$ *and a* $(1-\pi )100\%$ *PCI for* $z_{\tau :N},$ $\tau =s+1,s+2,\ldots ,N,$ *is* $\left[ \log \left( 1-\left( 1-e^{-\lambda z_{s:N}} \right) ^{(\psi +1)} \right) ^{\frac{-1}{\lambda }},z_{s:N} \right]$.

#### Proof

The JDF of $z_{s:N}$ and $z_{\tau :N}$, $1\le s<\tau \le N$, can be expressed as, see Raghunandanan and Patil^42^:

5.7 $$\begin{aligned} f_{s,\tau :N}^\star (x,y)=\frac{1}{\textrm{P}(N\ge \tau )}\sum ^{\infty }_{n=\tau }f_{s,\tau :n}^\star (x,y)\textrm{P}(N=n), \end{aligned}$$

where the JPDF $f_{s,\tau :n}^\star (x,y)$ is defined in (5.5). Using this JDF, we derive the JDF of the transformed variables $\rho _{s,\tau :N} = \log \left( 1 - e^{-\lambda z_{\tau :N}}\right) - \log \left( 1 - e^{-\lambda z_{s:N}}\right)$ and $\varrho _{s:N} = \log \left( 1 - e^{-\lambda z_{s:N}}\right)$ by applying the standard transformation method, then

5.8 $$\begin{aligned} f_{\rho _{s,\tau :N},\varrho _{s:N}}(\rho ,\varrho )= & \frac{1}{(s-1)!(\tau -s-1)!\textrm{P}(N\ge \tau )}\sum ^{\infty }_{n=s}\mathbf {\sum _{P}}\sum _{{\underline{l}},{\underline{i}},\underline{\textrm{v}},{\underline{h}},\underline{\nu }}^{s,\tau ,n}\frac{\textrm{Q}_{{\underline{l}},{\underline{i}},\underline{\textrm{v}},{\underline{h}},\underline{\nu }}(s,\tau ,n)}{(n-\tau )!} \nonumber \\\times & \theta ^{\sum _{\iota =1}^{s-1}l_{\iota }+\sum _{\iota =s+1}^{\tau -1}v_{\iota }+\sum _{\iota =\tau +1}^{n}\nu _{\iota }+i_{s}+i_{\tau }} \varrho ^{\sum _{\iota =1}^{s-1}l_{\iota }+\sum _{\iota =s+1}^{\tau -1}v_{\iota }(1-h_{\iota })+i_{r}-1}\nonumber \\\times & \left( \rho +\varrho \right) ^{\sum _{\iota =s+1}^{\tau -1}h_{\iota }v_{\iota }+\sum _{\iota =\tau +1}^{n}\nu _{\iota }+i_{\tau }-1} \nonumber \\\times & \exp \left[ \left( \left( \sum _{\iota =1}^{s-1}k_{\iota }+\sum _{\iota =s+1}^{\tau -1}k_{\iota }+\sum _{\iota =\tau +1}^{n}k_{\iota }+2k \right) \varrho \right. \right. \nonumber \\+ & \left. \left. \left( \sum _{\iota =s+1}^{\tau -1}k_{\iota }h_{\iota }+\sum _{\iota =\tau +1}^{n}k_{\iota }+k \right) \rho \right) \theta \right] \textrm{P}(N=n). \end{aligned}$$

Thus, the JDF of the pivotal quantity $\Psi ^{OMELRRSS}_{s,\tau :N} = \frac{\rho _{s,\tau :N}}{\varrho _{s:N}}$ and $\varrho _{s:N}$ is given by:

5.9 $$\begin{aligned} f_{\Psi ^{OMELRRSS}_{s,\tau :N},\varrho _{s:N}}(\psi ,\varrho )= & \frac{1}{(s-1)!(\tau -s-1)!\textrm{P}(N\ge \tau )}\sum ^{\infty }_{n=s}\mathbf {\sum _{P}}\sum _{{\underline{l}},{\underline{i}},\underline{\textrm{v}},{\underline{h}},\underline{\nu }}^{s,\tau ,n}\frac{\textrm{Q}_{{\underline{l}},{\underline{i}},\underline{\textrm{v}},{\underline{h}},\underline{\nu }}(s,\tau ,n)}{(n-\tau )!} \nonumber \\ \times & \theta ^{\sum _{\iota =1}^{s-1}l_{\iota }+\sum _{\iota =s+1}^{\tau -1}v_{\iota }+\sum _{\iota =\tau +1}^{n}\nu _{\iota }+i_{s}+i_{\tau }} \nonumber \\ \times & \varrho ^{\sum _{\iota =1}^{s-1}l_{\iota }+\sum _{\iota =s+1}^{\tau -1}v_{\iota }+\sum _{\iota =\tau +1}^{n}\nu _{\iota }+i_{s}+i_{\tau }-1} \nonumber \\ \times & \left( 1+\Psi \right) ^{\sum _{\iota =s+1}^{\tau -1}h_{\iota }v_{\iota }+\sum _{\iota =\tau +1}^{n}\nu _{\iota }+i_{\tau }-1} \nonumber \\ \times & \exp \left[ \left( \sum _{\iota =1}^{s-1}k_{\iota }+\sum _{\iota =s+1}^{\tau -1}k_{\iota }+\sum _{\iota =\tau +1}^{n}k_{\iota }+2k \right. \right. \nonumber \\+ & \left. \left. \left( \sum _{\iota =s+1}^{\tau -1}k_{\iota }h_{\iota }+\sum _{\iota =\tau +1}^{n}k_{\iota }+k \right) \Psi \right) \theta \varrho \right] \textrm{P}(N=n). \end{aligned}$$

Hence, the PDF of the pivotal quantity $\Psi ^{OMELRRSS}_{s,\tau :N}$ is given by:

5.10 $$\begin{aligned} f_{\Psi ^{OMELRRSS}_{s,\tau :N}}(\psi )= & \frac{1}{(s-1)!(\tau -s-1)!\textrm{P}(N\ge \tau )} \sum ^{\infty }_{n=\tau }\mathbf {\sum _{P}}\sum _{{\underline{l}},{\underline{i}},\underline{\textrm{v}},{\underline{h}},\underline{\nu }}^{s,\tau ,n}\textrm{Q}^{\star }_{{\underline{l}},{\underline{i}},\underline{\textrm{v}},{\underline{h}},\underline{\nu }}(s,\tau ,n)\nonumber \\ \times & \left( 1+\Psi \right) ^{V_{s,\tau ,n}(\theta )}\left( T_{s,\tau ,n}(\theta )+D_{s,\tau ,n}(\theta )\Psi \right) ^{-W_{s,\tau ,n}(\theta )}\textrm{P}(N=n), \end{aligned}$$

where $\textrm{Q}^{\star }_{{\underline{l}},{\underline{i}},\underline{\textrm{v}},{\underline{h}},\underline{\nu }}(s,\tau ,n)=\textrm{Q}_{{\underline{l}},{\underline{i}},\underline{\textrm{v}},{\underline{h}},\underline{\nu }}(s,\tau ,n)\theta ^{W_{s,\tau ,n}(\theta )}\Gamma (W_{s,\tau ,n}(\theta ))((n-\tau )!)^{-1}$, $W_{s,\tau ,n}(\theta )=\sum _{\iota =1}^{s-1}l_{\iota }+\sum _{\iota =s+1}^{\tau -1}v_{\iota }+\sum _{\iota =\tau +1}^{n}\nu _{\iota }+i_{s}+i_{\tau }$, $D_{s,\tau ,n}(\theta )=-\theta \left( \sum _{\iota =s+1}^{\tau -1}k_{\iota }h_{\iota }+\sum _{\iota =\tau +1}^{n}k_{\iota }+k\right)$, $V_{s,\tau ,n}(\theta )=\sum _{\iota =s+1}^{\tau -1}h_{\iota }v_{\iota }+\sum _{\iota =\tau +1}^{n}\nu _{\iota }+i_{s}-1$, $T_{s,\tau ,n}(\theta )=-\theta \left( \sum _{\iota =1}^{s-1}k_{\iota }+\sum _{\iota =s+1}^{\tau -1}k_{\iota }+\sum _{\iota =\tau +1}^{n}k_{\iota }+2k \right)$, $P(N=n)$ is the PMF of discrete RV $N$, and $\Upsilon (.)$ is hypergeometric 2F1 regularized function.

The SF, denoted as $\mathrm {\overline{F}_{\Psi ^{OMELRRSS}_{s,\tau :N}}}(\psi )$, is defined as: $\mathrm {\overline{F}_{\Psi ^{OMELRRSS}_{s,\tau :N}}}(\psi ) =P\left( \Psi ^{OMELRRSS}_{s,\tau :N} > \psi \right) .$ This function provides the probability that the pivotal quantity $\Psi ^{OMELRRSS}_{s,\tau :N}$ exceeds a given value $\psi$. After performing routine calculations, the SF of the pivotal quantity $\Psi ^{OMELRRSS}_{s,\tau :N}$ can be obtained. $\square$

#### Corollar 5.1

*According to Theorem* 5.1.1, *if N is a non-random integer, i.e.*, $P(N = n) = 1),~ n\ge \tau$, *the SF of* $\Psi ^{OMELRRSS}_{s,\tau :N}$ *takes the form*:

5.11 $$\begin{aligned} \mathrm {F_{\Psi ^{OMELRRSS}_{s,\tau :N}}}(\Psi )= & \left( (s-1)!(\tau -s-1)!(n-\tau )!\right) ^{-1}\mathbf {\sum _{P}}\sum _{{\underline{l}},{\underline{i}},\underline{\textrm{v}},{\underline{h}},\underline{\nu }}^{s,\tau ,n}\nonumber \\ \times & \frac{\textrm{Q}^{\star }_{{\underline{l}},{\underline{i}},\underline{\textrm{v}},{\underline{h}},\underline{\nu }}(s,\tau ,n)\Gamma (1+V_{s,\tau ,n}(\theta ))}{D_{s,\tau ,n}(\theta )-T_{s,\tau ,n}(\theta )}\left[ \left( T_{s,\tau ,n}(\theta )\right) ^{1-W_{s,\tau ,n}(\theta )} \right. \nonumber \\ \times & \left. \Upsilon \left( 1,2+V_{s,\tau ,n}(\theta )-W_{s,\tau ,n}(\theta ),2+V_{s,\tau ,n}(\theta ),\frac{D_{s,\tau ,n}(\theta )}{D_{s,\tau ,n}(\theta )-T_{s,\tau ,n}(\theta )}\right) \right. \nonumber \\ - & \left. \left( 1+\Psi \right) ^{1+V_{s,\tau ,n}(\theta )}\left( T_{s,\tau ,n}(\theta )+D_{s,\tau ,n}(\theta )\Psi \right) ^{1-W_{s,\tau ,n}(\theta )} \right. \nonumber \\ \times & \left. \Upsilon \left( 1,2+V_{s,\tau ,n}(\theta )-W_{s,\tau ,n}(\theta ),2+V_{s,\tau ,n}(\theta ),\frac{D_{s,\tau ,n}(\theta )\left( 1+\Psi \right) }{D_{s,\tau ,n}(\theta )-T_{s,\tau ,n}(\theta )}\right) \right] ,\; \Psi \ge 0,\nonumber \\ \end{aligned}$$

#### Corollar 5.2

*If *$N$ *follows a discrete uniform distribution with parameters* $\varsigma$ *and* $\kappa$ (*i.e.*, $N {\mathop {\sim }\limits ^{D}} \textrm{Uniform}(\kappa , \varsigma )$), *ihe SF of *$\Psi ^{OMELRRSS}_{s,\tau :N}$ *takes the form*:

5.12 $$\begin{aligned} \mathrm {F_{\Psi ^{OMELRRSS}_{s,\tau :N}}}(\Psi )= & \left( (s-1)!(\tau -s-1)!\textrm{P}(N\ge \tau )\right) ^{-1} \sum ^{\infty }_{n=\tau }\mathbf {\sum _{P}}\sum _{{\underline{l}},{\underline{i}},\underline{\textrm{v}},{\underline{h}},\underline{\nu }}^{s,\tau ,n}\nonumber \\ \times & \frac{\textrm{Q}^{\star }_{{\underline{l}},{\underline{i}},\underline{\textrm{v}},{\underline{h}},\underline{\nu }}(s,\tau ,n)\Gamma (1+V_{s,\tau ,n}(\theta ))}{(D_{s,\tau ,n}(\theta )-T_{s,\tau ,n}(\theta ))}\left[ \left( T_{s,\tau ,n}(\theta )\right) ^{1-W_{s,\tau ,n}(\theta )} \right. \nonumber \\ \times & \left. \Upsilon \left( 1,2+V_{s,\tau ,n}(\theta )-W_{s,\tau ,n}(\theta ),2+V_{s,\tau ,n}(\theta ),\frac{D_{s,\tau ,n}(\theta )}{D_{s,\tau ,n}(\theta )-T_{s,\tau ,n}(\theta )}\right) \right. \nonumber \\ - & \left. \left( 1+\Psi \right) ^{1+V_{s,\tau ,n}(\theta )}\left( T_{s,\tau ,n}(\theta )+D_{s,\tau ,n}(\theta )\Psi \right) ^{1-W_{s,\tau ,n}(\theta )} \right. \nonumber \\ \times & \left. \Upsilon \left( 1,2+V_{s,\tau ,n}(\theta )-W_{s,\tau ,n}(\theta ),2+V_{s,\tau ,n}(\theta ),\frac{D_{s,\tau ,n}(\theta )\left( 1+\Psi \right) }{D_{s,\tau ,n}(\theta )-T_{s,\tau ,n}(\theta )}\right) \right] \nonumber \\ \times & (\varsigma -\kappa +1)^{-1},\; \Psi \ge 0, \end{aligned}$$

*where* $$\textrm{P}(N\ge \tau )=\left\{ \begin{array}{ll} 1, \qquad \qquad \quad \textrm{if} \quad \tau \le \kappa , \\ \\ 1-\frac{\tau -\kappa }{\varsigma -\kappa +1},\quad \textrm{if} \quad \tau >\kappa . \end{array}\right.$$

## MC-simulation algorithm

An MC-simulation study is conducted to validate the theoretical results of estimation and prediction. Based on doubly type-II censored of upper OMELRRSS, the classical Bayes and empirical Bayes estimates of the unknown parameter $\theta$ are computed under a balanced loss function. These estimates are compared with the MLE for both fixed and random sample sizes $N$; see Algorithm 1. Additionally, the practical utility of the pivotal method, introduced in the previous section, is demonstrated for constructing an exact prediction interval for future order statistics $z_{\tau :n}$, where $\tau = s+1, \dots , n$. This interval is based on the observed order statistics (doubly type-II censored of lower OMELRRSS) $z_{r:n}> z_{r+1:n}> \dots > z_{s:n}$, with $r < s$; see Algorithm 2. The pivotal prediction method involves solving the nonlinear equation: $\mathrm {F_{\Psi ^{OMELRRSS}_{s,\tau :N}}}(\Psi ) = 1 - \pi ,$ to determine the value of $\Psi$. Numerical solutions to this equation are obtained using Mathematica 13, and simulation studies are performed for a significant lifetime distribution. Furthermore, the $100(1-\pi )\%$ PCI for $z_{\tau :n}$, the probability coverage (PC), interval length (IW), and the average interval width (AIW) are computed based on the statistic $\Psi ^{OMELRRSS}_{s,\tau :N}$ for both fixed and random sample sizes $N$.

**Algorithm 1:**
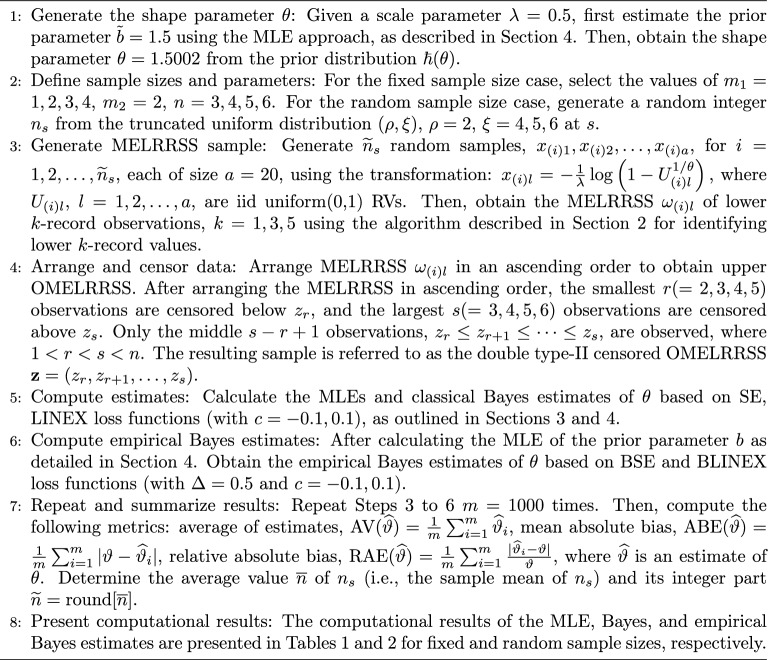
Generate Upper OMELRRSS for Estimation Procedures

**Table 1:** MLEs, Bayes estimates and empirical Bayes estimates of $\theta$ with their AVs, ABEs and RABs based on OMELRRSS, for different values of *n*, *r*, *s* and and *k*.

*k*	*n*	*r*	*s*	MLE	Bayes Estimate			Empirical Bayes Estimate		
						LINEX			BLINEX	
					SE	$c=0.1$	$c=-0.1$	BSE	$c=0.1$	$c=-0.1$
				AV	AV	AV	AV	AV	AV	AV
				ABE	ABE	ABE	ABE	ABE	ABE	ABE
				RAB	RAB	RAB	RAB	RAB	RAB	RAB
1	3	2	3	2.7660	1.2622	1.2399	1.2858	2.0141	1.8244	2.2013
				2.1149	0.6066	0.5849	0.6297	1.3608	1.1714	1.5476
				3.1647	0.9078	0.8752	0.9422	2.0362	1.7529	2.3158
	4	2	3	1.1378	0.8943	0.8839	0.9050	1.0160	1.0046	1.0273
				0.5673	0.2999	0.2916	0.3087	0.4332	0.4229	0.4434
				0.8497	0.4493	0.4367	0.4624	0.6489	0.6335	0.6641
		2	4	1.7225	1.2431	1.2281	1.2586	1.4828	1.4612	1.5039
				1.0654	0.5840	0.5693	0.5993	0.8247	0.8032	0.8456
				1.5981	0.8761	0.8541	0.8990	1.2371	1.2049	1.2685
		3	4	4.1886	1.8179	1.7863	1.8513	3.0032	2.6889	3.3120
				3.5219	1.1511	1.1195	1.1844	2.3365	2.0221	2.6452
				5.2770	1.7247	1.6773	1.7747	3.5008	3.0298	3.9634
	5	2	3	0.6677	0.6380	0.6332	0.6429	0.6528	0.6501	0.6556
				0.2452	0.1813	0.1801	0.1826	0.2128	0.2119	0.2136
				0.3677	0.2719	0.2701	0.2738	0.3191	0.3178	0.3204
		2	4	1.0434	0.9232	0.9159	0.9307	0.9833	0.9789	0.9877
				0.4218	0.2970	0.2907	0.3035	0.3594	0.3554	0.3633
				0.6328	0.4456	0.4362	0.4553	0.5392	0.5333	0.5450
		2	5	1.4160	1.2069	1.1973	1.2167	1.3115	1.3043	1.3185
				0.7564	0.5465	0.5371	0.5562	0.6515	0.6444	0.6585
				1.1372	0.8216	0.8074	0.8361	0.9793	0.9687	0.9898
		3	4	1.7454	1.2853	1.2706	1.3005	1.5154	1.4946	1.5356
				1.0860	0.6248	0.6103	0.6398	0.8554	0.8347	0.8755
				1.6305	0.9381	0.9163	0.9607	1.2843	1.2533	1.3145
		3	5	2.4981	1.7400	1.7195	1.7612	2.1190	2.0852	2.1516
				1.8317	1.0735	1.0530	1.0947	1.4526	1.4188	1.4852
				2.7467	1.6097	1.5791	1.6416	2.1782	2.1276	2.2271
		4	5	5.6267	2.5172	2.4754	2.5613	4.0719	3.6501	4.4844
				4.9604	1.8509	1.8091	1.8950	3.4057	2.9838	3.8181
				7.4449	2.7780	2.7152	2.8441	5.1114	4.4783	5.7305
	6	2	3	0.4579	0.4808	0.4783	0.4834	0.4694	0.4679	0.4709
				0.2515	0.2129	0.2139	0.2119	0.2319	0.2326	0.2312
				0.3775	0.3196	0.3211	0.3181	0.3481	0.3492	0.3471
		2	4	0.7207	0.7111	0.7072	0.7151	0.7159	0.7135	0.7183
				0.2192	0.1573	0.1553	0.1594	0.1878	0.1870	0.1885
				0.3282	0.2355	0.2325	0.2386	0.2811	0.2800	0.2822
		2	5	1.0729	0.9909	0.9852	0.9966	1.0319	1.0287	1.0351
				0.4286	0.3376	0.3323	0.3430	0.3825	0.3796	0.3853
				0.6431	0.5065	0.4986	0.5147	0.5739	0.5696	0.5781
		2	6	1.3323	1.2154	1.2088	1.2221	1.2738	1.2701	1.2775
				0.6693	0.5521	0.5455	0.5587	0.6107	0.6070	0.6143
				1.0040	0.8281	0.8183	0.8381	0.9160	0.9105	0.9215
		3	4	1.0345	0.9215	0.9145	0.9285	0.9780	0.9737	0.9822
				0.4145	0.2908	0.2848	0.2970	0.3524	0.3487	0.3560
				0.6222	0.4365	0.4275	0.4458	0.5289	0.5235	0.5344
		3	5	1.5100	1.2874	1.2772	1.2978	1.3987	1.3920	1.4052
				0.8492	0.6227	0.6126	0.6330	0.7357	0.7292	0.7422
				1.2750	0.9348	0.9197	0.9504	1.1046	1.0948	1.1143
		3	6	1.9996	1.6573	1.6441	1.6707	1.8284	1.8184	1.8383
				1.3337	0.9913	0.9782	1.0048	1.1625	1.1524	1.1724
				2.0024	1.4884	1.4687	1.5086	1.7454	1.7302	1.7602
		4	5	2.5995	1.8034	1.7827	1.8249	2.2015	2.1545	2.2471
				1.9323	1.1361	1.1154	1.1576	1.5342	1.4872	1.5798
				2.8948	1.7020	1.6710	1.7342	2.2984	2.2280	2.3667
		4	6	3.4455	2.3308	2.3041	2.3584	2.8881	2.8161	2.9579
				2.7796	1.6649	1.6383	1.6925	2.2222	2.1502	2.2920
				4.1743	2.5002	2.4603	2.5418	3.3373	3.2291	3.4420
		5	6	7.5229	3.3345	3.2813	3.3904	5.4287	4.7513	6.0909
				6.8567	2.6683	2.6152	2.7243	4.7625	4.0851	5.4248
				10.2930	4.0054	3.9256	4.0894	7.1490	6.1321	8.1431
3	3	2	3	2.8576	1.2445	1.2227	1.2675	2.0511	1.7842	2.3155
				2.2162	0.5968	0.5757	0.6191	1.4065	1.1401	1.6706
				3.3237	0.8951	0.8634	0.9285	2.1094	1.7098	2.5054
	4	2	3	1.0461	0.8513	0.8419	0.8611	0.9487	0.9397	0.9575
				0.4932	0.2780	0.2708	0.2855	0.3855	0.3777	0.3931
				0.7392	0.4166	0.4058	0.4279	0.5777	0.5661	0.5891
		2	4	1.7374	1.2265	1.2117	1.2419	1.4820	1.4519	1.5113
				1.0949	0.5799	0.5655	0.5948	0.8374	0.8076	0.8664
				1.6435	0.8704	0.8489	0.8928	1.2570	1.2123	1.3006
		3	4	4.3989	1.8275	1.7953	1.8615	3.1132	2.7274	3.4933
				3.7332	1.1613	1.1292	1.1952	2.4472	2.0615	2.8273
				5.5931	1.7399	1.6917	1.7907	3.6665	3.0885	4.2358
	5	2	3	0.5901	0.5792	0.5752	0.5832	0.5846	0.5825	0.5868
				0.2422	0.1927	0.1923	0.1933	0.2174	0.2170	0.2178
				0.3632	0.2891	0.2883	0.2900	0.3261	0.3255	0.3267
		2	4	0.9272	0.8449	0.8388	0.8512	0.8860	0.8825	0.8896
				0.3385	0.2483	0.2436	0.2532	0.2934	0.2906	0.2963
				0.5071	0.3720	0.3650	0.3793	0.4395	0.4353	0.4438
		2	5	1.3172	1.1436	1.1349	1.1524	1.2304	1.2246	1.2361
				0.6618	0.4865	0.4782	0.4951	0.5742	0.5686	0.5797
				0.9931	0.7301	0.7176	0.7429	0.8616	0.8532	0.8699
		3	4	1.6815	1.2518	1.2376	1.2665	1.4666	1.4495	1.4833
				1.0283	0.5966	0.5828	0.6109	0.8124	0.7955	0.8289
				1.5427	0.8951	0.8743	0.9166	1.2189	1.1935	1.2437
		3	5	2.3926	1.6759	1.6566	1.6960	2.0343	2.0002	2.0672
				1.7266	1.0098	0.9904	1.0299	1.3682	1.3342	1.4011
				2.6202	1.5338	1.5045	1.5643	2.0770	2.0279	2.1244
		4	5	5.8001	2.4991	2.4573	2.5432	4.1496	3.6607	4.6281
				5.1335	1.8326	1.7908	1.8766	3.4830	2.9941	3.9616
				7.7015	2.7493	2.6866	2.8154	5.2254	4.4919	5.9433
	6	2	3	0.3691	0.4159	0.4140	0.4178	0.3925	0.3912	0.3938
				0.3186	0.2643	0.2653	0.2632	0.2912	0.2921	0.2903
				0.4774	0.3960	0.3976	0.3944	0.4364	0.4378	0.4350
		2	4	0.6377	0.6383	0.6350	0.6415	0.6380	0.6360	0.6400
				0.2164	0.1667	0.1659	0.1676	0.1910	0.1908	0.1912
				0.3249	0.2503	0.2491	0.2517	0.2867	0.2864	0.2870
		2	5	0.9224	0.8751	0.8706	0.8797	0.8988	0.8963	0.9013
				0.3095	0.2460	0.2423	0.2498	0.2770	0.2751	0.2780
				0.4644	0.3690	0.3634	0.3748	0.4155	0.4127	0.4183
		2	6	1.1364	1.0615	1.0563	1.0670	1.0989	1.0961	1.1017
				0.4950	0.4127	0.4076	0.4179	0.4539	0.4510	0.4567
				0.7425	0.6190	0.6113	0.6268	0.6807	0.6765	0.6850
		3	4	0.9252	0.8446	0.8386	0.8506	0.8849	0.8812	0.8886
				0.3468	0.2433	0.2387	0.2480	0.2948	0.2920	0.2975
				0.5202	0.3649	0.3581	0.3719	0.4422	0.4380	0.4463
		3	5	1.4037	1.2082	1.1990	1.2176	1.3059	1.2996	1.3121
				0.7504	0.5500	0.5409	0.5593	0.6499	0.6438	0.6560
				1.1268	0.8258	0.8122	0.8397	0.9758	0.9665	0.9849
		3	6	1.7958	1.5202	1.5089	1.5318	1.6580	1.6495	1.6663
				1.1293	0.8535	0.8422	0.8651	0.9914	0.9829	0.9997
				1.6927	1.2794	1.2625	1.2968	1.4860	1.4733	1.4985
		4	5	2.4215	1.6893	1.6707	1.7087	2.0554	1.9943	2.1154
				1.7591	1.0154	0.9969	1.0345	1.3908	1.3297	1.4506
				2.6369	1.5335	1.5056	1.5625	2.0848	1.9932	2.1745
		4	6	3.2935	2.1964	2.1719	2.2218	2.7450	2.6397	2.8479
				2.6271	1.5300	1.5054	1.5554	2.0785	1.9733	2.1815
				3.9421	2.29508	2.259	2.3340	3.1189	2.9610	3.2734
		5	6	7.4710	3.2366	3.1858	3.2900	5.3538	4.5656	6.1278
				6.8049	2.5705	2.5197	2.6239	4.6877	3.8995	5.4617
				10.2160	3.8591	3.7829	3.9393	7.0377	5.8543	8.1997

### Discussion

From the estimation results tabulated in Tables 1 and 2, the following observations are made:

1. Effect of increasing $N$ and $s$: The AVs, ABEs, and ABs decrease as $N$ and $s$ increase for fixed values of $r$ and $k$.
2. Effect of increasing $\xi$, $k$, and $s$: The AVs, ABEs, and RABs increase as $\xi$, $k$, and $s$ increase for fixed values of $r$ and $N$.
3. Effect of increasing $r$ and $k$: The AVs, ABEs, and RABs increase as $r$ and $k$ increase for fixed values of $s$ and $N$.
4. Precision of results for $k = 3, 5$ vs. $k = 1$: The AVs, ABEs, and RABs for $k = 3, 5$ are generally more precise than those for $k = 1$ in most cases considered.
5. Effect of increasing $c$ in LINEX and BLINEX loss functions: The AVs, ABEs, and RABs for the LINEX and BLINEX loss functions decrease as $c$ increases for fixed values of $s$ and $n$.
6. Comparison of Bayes estimates with MLE and empirical Bayes estimates: The Bayes estimates of $\theta$ outperform both the MLE and empirical Bayes estimates in terms of AVs, ABEs, and RABs.
7. Comparison of empirical Bayes estimates with MLE: The empirical Bayes estimates of $\theta$ are superior to the MLEs in terms of AVs, ABEs, and RABs.
8. Comparison of LINEX-based Bayes estimates with BSE-based estimates: The Bayes estimates (classical Bayes) based on the LINEX loss function (with $c = 0.1$) outperform the Bayes estimates (empirical Bayes) based on the BSE loss function in terms of AVs, ABEs, and RABs.
9. Fixed vs. random sample size: A comparison of the estimation results for the fixed sample size case with those for the random sample size case shows that the former yields more precise results, as expected.
10. Comparison with existing results: The estimation results based on OMELRRSS are superior to those reported in^11,14^.
11. In the context of MLE, the Newton-Raphson algorithm follows specific convergence criteria to ensure numerical stability and efficiency:
  - Tolerance threshold: Convergence is achieved when the absolute difference between consecutive parameter estimates satisfies $|\theta _{k+1} - \theta _k| < \epsilon$ where $\epsilon$ is a predefined tolerance level, typically set to values such as $10^{-6}$.
  - Maximum iterations: To prevent infinite loops, a maximum number of iterations (e.g., 100) is enforced.
  - Initialization and stability: The algorithm starts with carefully chosen initial values, often derived from preliminary estimates, to enhance numerical stability and improve convergence behavior.

The numerical prediction results obtained from simulation studies, presented in Table 3, indicate the following:

1. Effect of increasing $\pi$, $s$, and $k$: The PCs and AIWs of the PCI for $z_{\tau }$ increase as $\pi$, $s$, and $k$ increase for fixed $\tau$ and $N$.
2. Effect of increasing $\tau$ and $N$: The PCs and AIWs of the PCI for $z_{\tau }$ decrease as $\tau$ and $N$ increase for fixed $s$ and $k$.
3. Effect of increasing $\xi$: The PCs and AIWs of the PCI for $z_{\tau }$ increase as the parameter $\xi$ increases for fixed $\kappa$.
4. Fixed vs. random sample sizes: The PCs and AIWs of the PCIs for $z_{\tau }$ based on fixed sample sizes are better than those based on random sample sizes.
5. Precision of results for $k = 1$ vs. $k = 3, 5, 7$: In most cases, the probability coverage deviates significantly from $1 - \pi$. The results for $k = 1$ are more precise than those for $k = 3, 5, 7$ across all cases considered.
6. Comparison with existing methods: In all cases, the prediction results based on upper non-identical order statistics of the upper $k$-record of MERSS outperform those based on lower non-identical order statistics of the lower $k$-record of ERSS, as shown in^13^.

Based on the above observations, we recommend using the classical Bayes approach for parameter estimation and the pivotal prediction method incorporated in the double type-II censoring of OMELRRSS.

**Algorithm 2:**
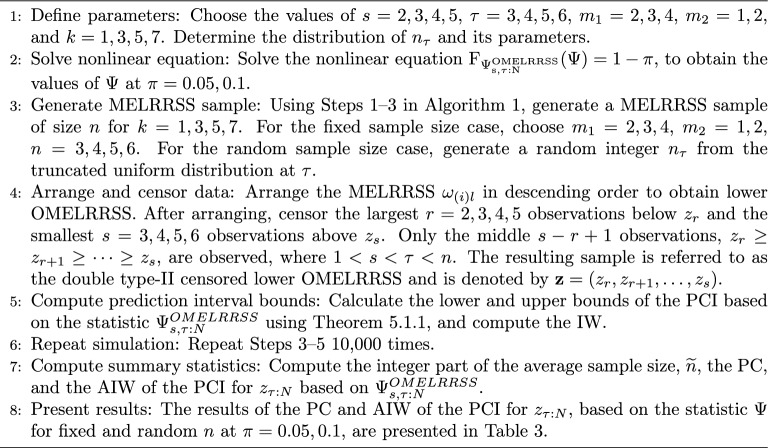
Generate Lower OMELRRSS for Pivotal Prediction Procedure

**Table 2:** MLEs, Bayes estimates and empirical Bayes estimates of $\theta$ with their AVs, ABEs and RABs, respectively based on OMELRRSS, for different values of $\xi$, *r*, *s* and *k*.

${\widetilde{n}}[\xi ]$	*r*	*s*	*k*	MLE	Bayes Estimate			Empirical Bayes Estimate		
						LINEX			BLINEX	
					SE	$c=0.1$	$c=-0.1$	BSE	$c=0.1$	$c=-0.1$
				AV	AV	AV	AV	AV	AV	AV
				ABE	ABE	ABE	ABE	ABE	ABE	ABE
				RAB	RAB	RAB	RAB	RAB	RAB	RAB
4[4]	2	3	1	2.0288	0.9216	0.9098	0.9338	1.4752	1.4337	1.5163
				1.3837	0.3192	0.3095	0.3295	0.8244	0.7833	0.8651
				2.0764	0.4790	0.4644	0.4945	1.2372	1.1755	1.2983
4[5]	2	3	1	2.5457	0.8078	0.7985	0.8175	1.6768	1.5961	1.7570
				1.8968	0.2517	0.2453	0.2586	1.0245	0.9440	1.1045
				2.8423	0.3772	0.3676	0.3875	1.5352	1.4146	1.6550
5[5]	2	4	1	1.5820	0.9506	0.9425	0.9589	1.2663	1.2479	1.2846
				0.9290	0.3206	0.3135	0.3280	0.6125	0.5943	0.6306
				1.3946	0.4813	0.4706	0.4923	0.9194	0.8921	0.9466
	3	4	1	2.2448	1.3365	1.3195	1.3541	1.7906	1.7533	1.8272
				1.5798	0.6740	0.6573	0.6915	1.1254	1.0881	1.1619
				1.5798	1.0107	0.9856	1.0368	1.6873	1.6314	1.7421
4[6]	2	3	1	3.1419	0.7317	0.7238	0.7398	1.9368	1.7174	2.1557
				2.4897	0.2344	0.2300	0.2390	1.2821	1.0629	1.5008
				3.7387	0.3520	0.3454	0.3590	1.9253	1.5962	2.2537
5[6]	2	4	1	2.0740	0.8482	0.8418	0.8548	1.4611	1.4149	1.5071
				1.4218	0.2445	0.2395	0.2497	0.8081	0.7621	0.8539
				2.1356	0.3673	0.3598	0.3750	1.2138	1.1447	1.2826
6[6]	2	5	1	1.3293	0.9771	0.9714	0.9828	1.1532	1.1433	1.1631
				0.6720	0.3256	0.3203	0.3310	0.4943	0.4846	0.5041
				1.0079	0.4884	0.4805	0.4964	0.7414	0.7268	0.7560
5[6]	3	4	1	2.5973	1.1504	1.1377	1.1637	1.8739	1.7967	1.9505
				1.9296	0.4955	0.4833	0.5083	1.2061	1.1289	1.2826
				2.8882	0.7418	0.7234	0.7609	1.8053	1.6898	1.9199
6[6]	3	5	1	1.7473	1.3089	1.2980	1.3201	1.5281	1.5154	1.5406
				1.0837	0.6451	0.6343	0.6562	0.8629	0.8502	0.8753
				1.6257	0.9678	0.9516	0.9845	1.2945	1.2756	1.3132
6[6]	4	5	1	2.7106	1.8133	1.7915	1.8360	2.2620	2.1966	2.3260
				2.0434	1.1461	1.1243	1.1688	1.5947	1.5293	1.6587
				3.0623	1.7176	1.6849	1.7516	2.3900	2.2920	2.4859
4[4]	2	3	3	1.7963	0.9034	0.8915	0.9158	1.3499	1.3210	1.3783
				1.1637	0.3010	0.2917	0.3109	0.7110	0.6827	0.7390
				1.7455	0.4515	0.4375	0.4664	1.0665	1.0240	1.1085
4[5]	2	3	3	2.2991	0.8238	0.8133	0.8348	1.5615	1.4601	1.6622
				1.6674	0.2550	0.2479	0.2627	0.9227	0.8218	1.0231
				2.5006	0.3825	0.3718	0.3940	1.3839	1.2326	1.5343
5[5]	2	4	3	1.6437	0.9183	0.9102	0.9267	1.2810	1.2601	1.3018
				0.9959	0.2924	0.2855	0.2996	0.6314	0.6107	0.6518
				1.4932	0.4384	0.4281	0.4492	0.9466	0.9156	0.9773
5[5]	3	4	3	2.0957	1.2624	1.2465	1.2790	1.6790	1.6477	1.7098
				1.4354	0.6053	0.5899	0.6215	1.0176	0.9863	1.0482
				2.1528	0.9079	0.8847	0.9322	1.5261	1.4792	1.5721
5[6]	2	3	3	2.4643	0.7040	0.6956	0.7127	1.5842	1.4733	1.6946
				1.8294	0.2124	0.2091	0.2159	0.9447	0.8342	1.0548
				2.7479	0.3190	0.3141	0.3244	1.4191	1.2531	1.5844
5[6]	2	4	3	1.8087	0.7867	0.7805	0.7930	1.2977	1.2676	1.3277
				1.1745	0.2131	0.2091	0.2173	0.6599	0.6301	0.6895
				1.7617	0.3196	0.3136	0.3260	0.9898	0.9451	1.0343
6[6]	2	5	3	1.4129	0.8781	0.8732	0.8831	1.1455	1.1331	1.1578
				0.7672	0.2479	0.2439	0.2521	0.4972	0.4851	0.5093
				1.1509	0.3720	0.3658	0.3783	0.7459	0.7277	0.7640
5[6]	3	4	3	2.4448	1.1444	1.1308	1.1587	1.7946	1.7005	1.8880
				1.7831	0.4930	0.4801	0.5065	1.1318	1.0378	1.2251
				2.6751	0.7396	0.7203	0.7599	1.6980	1.5570	1.8380
6[6]	3	5	3	1.7564	1.2405	1.2300	1.2513	1.4984	1.4837	1.5130
				1.0946	0.5790	0.5686	0.5896	0.8350	0.8203	0.8494
				1.6414	0.8682	0.8527	0.8841	1.2521	1.2301	1.2738
6[6]	4	5	3	2.5104	1.7097	1.6895	1.7308	2.1100	2.0785	2.1407
				1.8449	1.0442	1.0240	1.0654	1.4445	1.4129	1.4751
				2.7710	1.5685	1.5381	1.6002	2.1855	2.1180	2.2514
4[4]	2	3	5	1.8141	0.9346	0.9218	0.9480	1.3743	1.3462	1.4021
				1.1775	0.3227	0.3122	0.3340	0.7336	0.7060	0.7608
				1.7659	0.4840	0.4682	0.5009	1.1003	1.0589	1.1411
4[5]	2	3	5	2.3180	0.8374	0.8265	0.8489	1.5777	1.4577	1.6973
				1.6827	0.2592	0.2517	0.2672	0.9383	0.8187	1.0575
				2.5286	0.3895	0.3783	0.4016	1.4102	1.2304	1.5892
5[5]	2	4	5	1.5231	0.9037	0.8957	0.9121	1.2134	1.1976	1.2291
				0.8848	0.2914	0.2848	0.2982	0.5722	0.5568	0.5876
				1.3278	0.4373	0.4274	0.4475	0.8587	0.8355	0.8817
5[5]	3	4	5	2.1290	1.2960	1.2793	1.3135	1.7128	1.6782	1.7466
				1.4657	0.6340	0.6175	0.6512	1.0482	1.0137	1.0820
				2.1981	0.9508	0.9262	0.9766	1.5720	1.5203	1.6227
4[6]	2	3	5	2.4034	0.7208	0.7119	0.7302	1.5621	1.4596	1.6641
				1.7681	0.2009	0.1972	0.2050	0.9212	0.8192	1.0228
				2.6504	0.3011	0.2956	0.3073	1.3810	1.2281	1.5331
5[6]	2	4	5	1.7247	0.7929	0.7863	0.7996	1.2588	1.2325	1.2849
				1.0979	0.2254	0.2211	0.2299	0.6280	0.6021	0.6537
				1.6446	0.3376	0.3312	0.3444	0.9407	0.9019	0.9793
6[6]	2	5	5	1.3743	0.8833	0.8782	0.8886	1.1288	1.1175	1.1400
				0.7332	0.2540	0.2497	0.2584	0.4835	0.4725	0.4944
				1.0988	0.3806	0.3741	0.3872	0.7245	0.7081	0.7408
5[6]	3	4	5	2.2019	1.1170	1.1037	1.1308	1.6594	1.5986	1.7197
				1.5430	0.4627	0.4501	0.4759	0.9988	0.9381	1.0590
				2.3150	0.6942	0.6753	0.7141	1.4985	1.4075	1.5889
6[6]	3	5	5	1.7402	1.2306	1.2201	1.2414	1.4854	1.4717	1.4989
				1.0779	0.5669	0.5566	0.5776	0.8214	0.8078	0.8349
				1.6187	0.8514	0.8359	0.8674	1.2336	1.2132	1.2538
6[6]	4	5	5	2.5321	1.7105	1.6902	1.7317	2.1213	2.0764	2.1652
				1.8662	1.0446	1.0243	1.0658	1.4554	1.4104	1.4993
				2.8024	1.5686	1.5381	1.6004	2.1855	2.1180	2.2514

**Table 3:** PC and AIW based on statistic $\Psi$ with different values of *n*, $\xi$, $\pi$ and *k*.

${\widetilde{n}}[\xi ]$	*s*	$\tau$	$\pi$	$k=1$		$k=3$		$k=5$		$k=7$	
				PC	AIW	PC	AIW	PC	AIW	PC	AIW
F3	2	3	0.05	0.7639	0.6328	0.7660	0.9673	0.7638	1.0721	0.7576	1.1248
			0.10	0.7855	0.6542	0.7894	1.0116	0.7867	1.1255	0.7820	1.1830
4	2	3	0.05	0.9878	0.8899	0.9860	1.6220	0.9875	1.9234	0.9835	2.0956
			0.10	0.9888	0.8928	0.9876	1.6357	0.9890	1.9458	0.9854	2.1246
4[4]	2	3	0.05	0.9240	0.7889	0.9227	1.2503	0.9202	1.4056	0.9237	1.4779
			0.10	0.9321	0.8025	0.9306	1.2851	0.9309	1.4502	0.9325	1.5277
4	2	4	0.05	0.8301	0.8502	0.8209	1.4324	0.8242	1.6458	0.8212	1.7572
			0.10	0.8454	0.8577	0.8354	1.4565	0.8383	1.6797	0.8371	1.7970
	3	4	0.05	0.9645	0.4516	0.9611	1.0214	0.9613	1.2729	0.9615	1.4164
			0.10	0.9696	0.4542	0.9662	1.0372	0.9673	1.2997	0.9664	1.4508
5	2	3	0.05	0.9985	0.9352	0.9971	1.7411	0.9973	2.1545	0.9971	2.4117
			0.10	0.9987	0.9358	0.9973	1.7452	0.9975	2.1632	0.997	2.4252
4[5]	2	3	0.05	0.9847	0.9039	0.9843	1.5836	0.9813	1.8628	0.9808	2.0154
			0.10	0.9861	0.9078	0.9870	1.6001	0.9828	1.8889	0.9834	2.0482
5	2	4	0.05	0.9806	0.9388	0.9758	1.7118	0.9740	2.0689	0.9784	2.2738
			0.10	0.9835	0.9406	0.9782	1.7216	0.9774	2.0871	0.9811	2.2989
5[5]	2	4	0.05	0.9801	0.9382	0.9784	1.7064	0.9774	2.0653	0.9790	2.2768
			0.10	0.9835	0.9400	0.9813	1.7161	0.9804	2.0834	0.9810	2.3021
5	2	5	0.05	0.9690	0.9462	0.9618	1.7563	0.9580	2.1412	0.9602	2.3805
			0.10	0.9728	0.9470	0.9663	1.7619	0.9625	2.1522	0.9645	2.3968
5	3	4	0.05	1.0000	0.5038	0.9999	1.2215	0.9999	1.6403	1.0000	1.9278
			0.10	1.0000	0.5039	0.9999	1.2222	0.9999	1.6425	1.0000	1.9321
5[5]	3	4	0.05	0.9973	0.5120	0.9965	1.1814	0.9969	1.5273	0.9960	.7437
			0.10	0.9976	0.5126	0.9971	1.1872	0.9972	1.5403	0.9960	1.7634
5	3	5	0.05	0.9981	0.5025	0.9961	1.2139	0.9967	1.6211	0.9970	1.9072
			0.10	0.9984	0.5026	0.9961	1.2139	0.9972	1.6244	0.9974	1.9137
5	4	5	0.05	0.9976	0.2513	0.9986	0.7878	0.9975	1.1274	0.9967	1.3623
			0.10	0.9980	0.2514	0.9989	0.7902	0.9981	1.1350	0.9974	1.3762
6	2	3	0.05	0.9993	0.9469	0.9993	1.7900	0.9993	2.2446	0.9991	2.5165
			0.10	0.9994	0.9472	0.9995	1.7917	0.9993	2.2484	0.9991	2.5226
4[6]	2	3	0.05	0.9953	0.9017	0.9949	1.6986	0.9941	2.0680	0.9952	2.2749
			0.10	0.9960	0.9031	0.9956	1.7065	0.9947	2.0832	0.9959	2.2957
6	2	4	0.05	0.9976	0.9309	0.9970	1.7863	0.9972	2.2282	0.9975	2.5122
			0.10	0.9976	0.9310	0.9970	1.7871	0.9974	2.2301	0.9976	2.5154
5[6]	2	4	0.05	0.9971	0.9228	0.9965	1.7856	0.9969	2.2180	0.9971	2.4950
			0.10	0.9972	0.9229	0.9967	1.7864	0.9969	2.2199	0.9974	2.4981
6	2	5	0.05	0.9992	0.9234	0.9986	1.8108	0.9995	2.2502	0.9993	2.5826
			0.10	0.9992	0.9234	0.9989	1.8110	0.9995	2.2507	0.9993	2.5836
6[6]	2	5	0.05	0.9883	0.9219	0.9843	1.7672	0.9844	2.1795	0.9819	2.4577
			0.10	0.9896	0.9224	0.9856	1.7706	0.9863	2.1870	0.9836	2.4697
6	2	6	0.05	0.9987	0.9217	0.9967	1.8117	0.9966	2.2667	0.9966	2.5873
			0.10	0.9987	0.9217	0.9968	1.8118	0.9967	2.2668	0.9967	2.5876
6	3	4	0.05	1.0000	0.4958	1.0000	1.2291	1.0000	1.6542	1.0000	1.9528
			0.10	1.0000	0.4958	1.0000	1.2295	1.0000	1.6544	1.0000	1.9532
5[6]	3	4	0.05	0.9995	0.4882	0.9996	1.2214	0.9998	1.6388	0.9999	1.9247
			0.10	0.9997	0.4882	0.9997	1.2221	0.9998	1.6411	0.9999	1.9293
6	3	5	0.05	0.9998	0.4975	0.9999	1.2252	0.9999	1.6628	1.0000	1.9536
			0.10	1.0000	0.4975	0.9999	1.2253	1.0000	1.6632	1.0000	1.9546
6[6]	3	5	0.05	0.9980	0.5031	0.9971	1.2181	0.9964	1.6173	0.9962	1.8772
			0.10	0.9988	0.5035	0.9987	1.2240	0.9979	1.6356	0.9980	1.9103
6	3	6	0.05	0.9999	0.4924	0.9999	1.2318	0.9999	1.6624	0.9998	1.9640
			0.10	0.9999	0.4924	0.9999	1.2318	0.9999	1.6625	0.9999	1.9644
6	4	5	0.05	1.0000	0.2554	1.0000	0.8327	1.0000	1.2159	1.0000	1.5019
			0.10	1.0000	0.2554	1.0000	0.8328	1.0000	1.2160	1.0000	1.5020
6[6]	4	5	0.05	1.0000	0.2529	1.0000	0.8281	1.0000	1.2135	1.0000	1.4843
			0.10	1.0000	0.2529	1.0000	0.8283	1.0000	1.2145	1.0000	1.4869
6	4	6	0.05	1.0000	0.2586	1.0000	0.8233	1.0000	1.2181	1.0000	1.5025
			0.10	1.0000	0.2586	1.0000	0.8233	1.0000	1.2181	1.0000	1.5026
6	5	6	0.05	1.0000	0.1207	1.0000	0.5283	0.9999	0.8620	0.9999	1.1090
			0.10	1.0000	0.1207	1.0000	0.5285	1.0000	0.8629	0.9999	1.1118

## Data analysis

This section presents two real-life datasets to illustrate the practical application of the theoretical concepts discussed in this research.

**Fig. 2:**
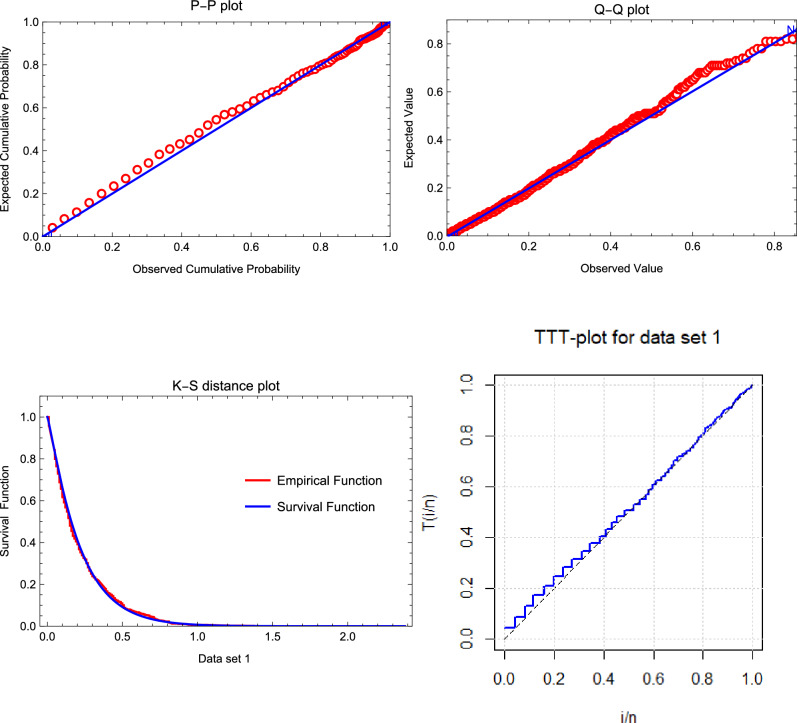
P-P, Q-Q, K-S distance, and TTT plots for data set 1.

**Fig. 3:**
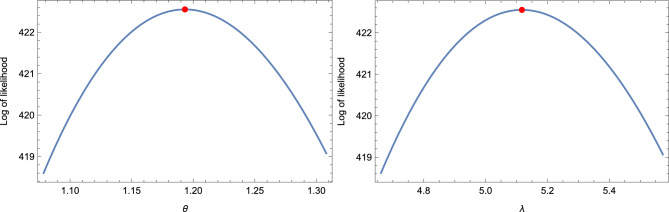
The log-likelihood functions of $\theta$ and $\lambda$ for data set 1.

**Data set 1** (Nerve Impulse Times). The first data set, provided in [^43^, pp. 124], consists of 800 recorded waiting times representing the time intervals between consecutive pulses along a nerve fiber, measured in seconds. The Kolmogorov-Smirnov (K-S) statistic and its corresponding p-value for this data set are $1.7410$ and $0.1281$, respectively. These values are based on the empirical CDF of $y_1, y_2, \ldots , y_n$, defined as: $\textrm{F}_i(y) = \frac{1}{n} \sum _{i=1}^n \mathrm {I_{1}}(y_i \le y),$ where $\mathrm {I_{1}}(\cdot )$ is the indicator function. Figure 2 displays the P-P plot, Q-Q plot, K-S distance plot, and total test time (TTT) plot for this data set. The K-S statistic, p-value, P-P plot, Q-Q plot, and K-S distance plot in Figure 2 suggest that the GED provides a good fit for these data. The failure rate function of a random variable $\textrm{T}$ can exhibit various patterns. To empirically analyze the failure rate behavior, the TTT plot, introduced by Aarset^44^, is used. The TTT plot can take on several shapes, providing insights into the failure rate patterns. Aarset^44^ demonstrated that if the curve approaches a straight diagonal function, a constant failure rate is adequate. The TTT curve is formed with values *r*/*n* and *G*(*r*/*n*), where $G(r/n) = \frac{\sum _{i=1}^{r}\textrm{T}_{i:n}+(n-r)\textrm{T}_{r:n}}{\sum _{i=1}^{n}\textrm{T}_{i:n}}, r=1,\ldots ,n,$ and $T_{1:n}=1,\ldots ,n.$ As a result, the TTT plot shows that the GED distribution is the best fit for the data set. Figure 3 depicts the log-likelihood functions of $\theta$ and $\lambda$, demonstrating that the MLEs are unique and exist.

**Fig. 4:**
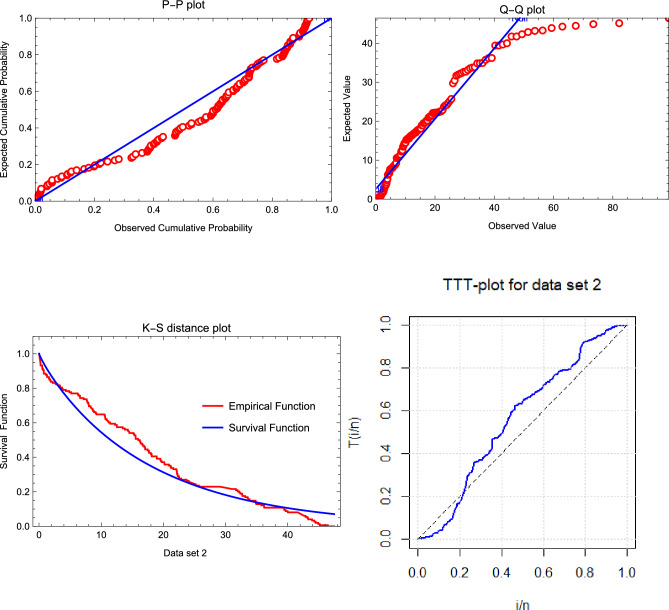
P-P, Q-Q, K-S distance, and TTT plots for data set 2.

**Fig. 5:**
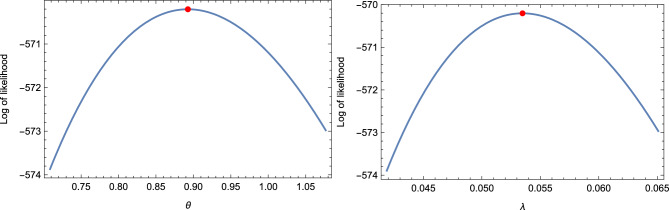
The log-likelihood functions of $\theta$ and $\lambda$ for data set 2.

**Data set 2** (Renal Transplant Data). The second data set, reported in [^43^, pp. 369–370], consists of the graft survival times (in months) of 148 renal transplant patients. For this data set, the K-S statistic and its corresponding p-value are $0.2655$ and $0.1695$, respectively. Figure 4 displays the P-P plot, Q-Q plot, K-S distance plot, and TTT plot for this data set. The K-S statistic, p-value, P-P plot, Q-Q plot, and K-S distance plot collectively demonstrate that the GED is a suitable model for fitting these data. The TTT plot further supports the suitability of the GED distribution for modeling this data set. Figure 5 illustrates the log-likelihood functions of $\theta$ and $\lambda$, confirming that the MLEs based on these data are unique. We note that the prior parameter *b* in our empirical Bayes framework was estimated directly from the data using the method outlined in Section 4, which maximizes the marginal likelihood. For Data set 1, $\lambda = 1.5$ and ${\tilde{b}} = 0.3672$, yielding $\theta = 2.7220$ from Eq. (4.1). Similarly, for Data set 2, $\lambda = 1.5$ and ${\tilde{b}} = 0.2312$, leading to $\theta = 4.3225$ from Eq. (4.1).

According to the MELRRSS scheme, seven SRSs ($n = 7, m_1 = 5, m_2 = 2$) of lower $k(= 1, 3, 5)$-record values is obtained for the two datasets: Data Set 1 ($a=100$) and Data Set 2 ($a=20$). The $i$-th lower $k$-record is selected from each of the first $m_1$ chosen sets, where $i = 1, \ldots , m_1$. Subsequently, the largest lower $k$-record is chosen from the remaining $m_2$ sets. The results of the MELRRSS for both datasets, with three different lower $k$-record values ($k = 1, 3, 5$), are presented in Tables 4 and 5. Sticking to the OMELRRSS process described in Section 2, the two real datasets are subjected to double type-II censoring of upper OMELRRSS, as shown in Table 6. Based on the samples in Table 6, the following estimates of the parameter $\theta$ are computed: MLEs, classical Bayes estimates, and empirical Bayes estimates based on: BSE, BLINEX loss functions (with $\Delta = 0.5$ and $c = -0.1, 0.1$). These estimates are calculated for fixed sample size $n = 7$, and random sample size $n_{s}$ (truncated uniform distribution at $s = 4, 5, 6, 7$, with parameters $\rho = 2$ and $\xi = 7$). The estimates results are computed for different $k$-record values and are presented in Table 7.

Assuming a double type-II censoring of lower OMELRRSS, where $s - r + 1$ failures are observed, prediction intervals for the unobserved failures $z_{\tau }$ ($\tau = s+1, \ldots , n_{\tau }$) are constructed based on the statistic $\Psi ^{OMELRRSS}_{s,\tau :n_{\tau }}$. The quantile values for $\pi = 0.05$ and $0.10$ are used to obtain these intervals. Specifically, we forecast the last $(n_{\tau } - \tau )$ observations for $\tau = s, \ldots , n_{\tau }$, based on the $s - r + 1$ observations with $r = 2, s=4$. This is done for fixed sample size $n = 7$, and random sample size $n_{\tau }$, where $n_{\tau } {\mathop {\sim }\limits ^{D}} \textrm{Uniform}(2, 7)$. Table 8 presents the quantile values (for $\pi = 0.05, 0.10$), PCIs for $z_{\tau :n_{\tau }}$ ($\tau = s, \ldots , n_{\tau }$), and IWs based on data sets 1 and 2, respectively.

The preceding tables provide a complete picture of the parameter estimations under various double type-II censoring of OMELRRSS, allowing insight into the performance of various estimation and prediction methodologies. The results show that the suggested technique is both applicable and successful, allowing for a complete comprehension of the GED distribution in the setting of double type-II censoring of OMELRRSS. Table 7 shows that Bayes and empirical Bayes estimates under BSE and BLINEX (with $c=-0.1$) perform better than MLEs. In all cases, the real values of $z_{\tau :N},$ $\tau = 4,5,6$ were found in the PCIs for the two data sets. Finally, estimations and predictions based on fixed sample sizes outperform the random assumptions in all cases.

## Concluding remarks

This study introduced a novel sampling strategy-ordered moving extremes lower k-record ranked set sampling-that significantly enhances parameter estimation and prediction for the generalized exponential distribution. By developing maximum likelihood, classical Bayes, and empirical Bayes estimators (under both symmetric and asymmetric loss functions), the methodology not only achieves lower mean squared errors and reduced biases but also provides robust prediction intervals through a pivotal approach. Extensive simulation studies and real-world applications in reliability and survival analysis confirm that the proposed method improves estimation efficiency compared to conventional techniques. Moreover, the paper demonstrates the versatility of OMELRRSS by incorporating random sample sizes, which better reflects practical data collection scenarios. This innovation opens new avenues for applying advanced statistical inference in high-cost and resource-constrained environments. However, the study also faces certain limitations, such as the complexity of the derived estimators and the reliance on iterative numerical methods (e.g., Newton-Raphson) to solve non-linear equations, which may challenge its practical implementation in some settings. Future work should aim to simplify these computational procedures and explore extensions to other lifetime distributions. Overall, this research makes a valuable contribution to the growing body of knowledge in statistical inference and reliability studies, offering both theoretical insights and practical tools for improved data analysis.

**Table 4:** Lower k-records values, their inter-record times, and lower MELRRSS, extracted from data set 1.

k = 1	$\underline{0.08}(1)$					$\rightarrow$	0.08
	0.29(1)	$\underline{0.05}(5)$				$\rightarrow$	0.05
	0.04(1)	0.02(2)	0.01(8)			$\rightarrow$	0.01
	0.05(1)	0.04(2)	0.03(11)	0.02(39)		$\rightarrow$	0.02
	0.24(1)	0.07(2)	0.03(5)	0.02(7)	0.01(26)	$\rightarrow$	0.01
	0.12(1)					$\rightarrow$	0.12
	0.59(1)	0.27(2)				$\rightarrow$	0.59
k = 3	0.38(3)					$\rightarrow$	0.38
	0.74(3)	0.31(4)				$\rightarrow$	0.31
	0.16(3)	0.08(4)	0.07(5)			$\rightarrow$	0.07
	0.10(3)	0.09(4)	0.05(11)	0.04(33)		$\rightarrow$	0.04
	0.24(3)	0.21(4)	0.17(5)	0.07(7)	0.06(16)	$\rightarrow$	0.06
	0.43(3)					$\rightarrow$	0.43
	0.59(3)	0.27(4)				$\rightarrow$	0.59
k = 5	0.41(5)					$\rightarrow$	0.41
	0.74(5)	0.31(6)				$\rightarrow$	0.31
	0.16(5)	0.09(6)	0.08(8)			$\rightarrow$	0.08
	0.13(5)	0.10(6)	0.09(7)	0.08(11)		$\rightarrow$	0.08
	0.24(5)	0.21(7)	0.17(8)	0.13(10)	0.12(15)	$\rightarrow$	0.12
	0.43(5)					$\rightarrow$	0.43
	0.59(5)	0.27(7)				$\rightarrow$	0.59

*(.) denote the inter-record times of lower k-record values

**Table 5:** Lower k-records values, their inter-record times, and lower MELRRSS, extracted from data set 2.

k = 1	$\underline{0.533}(1)$					$\rightarrow$	0.533
	4.867(1)	$\underline{1.600}(2)$				$\rightarrow$	1.600
	0.167(1)	0.101(3)	0.068(15)			$\rightarrow$	0.068
	1.639(1)	0.533(2)	0.233(8)	0.035(10)		$\rightarrow$	0.035
	3.700(1)	1.267(2)	0.633(4)	0.101(6)	0.068(11)	$\rightarrow$	0.068
	$\underline{2.967}(1)$					$\rightarrow$	2.967
	$\underline{1.639}(1)$	0.168(3)				$\rightarrow$	1.639
k = 3	$\underline{0.533}(3)$					$\rightarrow$	0.533
	4.867(3)	1.803(4)				$\rightarrow$	1.803
	0.770(3)	0.767(6)	0.508(8)			$\rightarrow$	0.508
	1.803(3)	1.639(5)	1.066(8)	0.533(10)		$\rightarrow$	0.533
	3.803(3)	3.700(4)	1.600(5)	1.267(6)	0.633(8)	$\rightarrow$	0.633
	$\underline{3.328}(3)$					$\rightarrow$	3.328
	$\underline{2.967}(3)$	1.867(4)				$\rightarrow$	2.967
k = 5	$\underline{2.180}(5)$					$\rightarrow$	2.180
	4.867(5)	3.700(6)				$\rightarrow$	3.700
	3.700(5)	1.600(6)	0.770(8)			$\rightarrow$	0.770
	1.867(5)	1.803(8)	1.639(9)	1.300(10)		$\rightarrow$	1.300
	3.803(5)	3.700(6)	1.600(8)	1.267(9)	1.066(11)	$\rightarrow$	1.066
	$\underline{3.700}(5)$					$\rightarrow$	3.700
	$\underline{2.967}(5)$	1.867(9)				$\rightarrow$	2.967

*(.) denote the inter-record times of lower k-record values

**Table 6:** Lower OMELRRSS based on data sets 1 and 2, respectively.

Data set	k							
1	1	0.5900	0.1200	0.0800	0.0500	0.0200	0.0100	0.0100
	3	0.5900	0.4300	0.3800	0.3100	0.0700	0.0600	0.0400
	5	0.5900	0.4300	0.4100	0.3100	0.1200	0.0800	0.0800
2	1	2.9670	1.6390	1.6000	0.5330	0.0680	0.0680	0.0350
	3	3.3280	2.9670	1.8030	0.6330	0.5330	0.5330	0.5080
	5	3.7000	3.7000	2.9670	2.1800	1.3000	1.0660	0.7700

**Table 7:** Based on the data sets 1 and 2 given in Table 6, MLEs, Bayes estimates and empirical Bayes estimates of $\theta$ based on OMELRRSS of fixed (Case I) and random sample sizes (Case II).

Data set	*k*	Case	*r*	*s*	MLE	Bayes Estimate			Empirical Bayes Estimate		
						SE	LINEX		BSE	BLINEX	
							$c=0.1$	$c=-0.1$		$c=0.1$	$c=-0.1$
1	1	I	3	5	0.9045	0.8708	0.8673	0.8745	0.8877	0.8858	0.8895
		II		4	0.7000	0.7186	0.7145	0.7228	0.7093	0.7072	0.7114
				5	0.8282	0.8149	0.8116	0.8182	0.8216	0.8199	0.8232
				6	1.0287	0.9907	0.9872	0.9944	1.0097	1.0079	1.0116
				7	1.1743	1.1291	1.1254	1.1328	1.1517	1.1498	1.1536
	3	I	3	5	0.3283	0.3413	0.3408	0.3419	0.3348	0.3345	0.3351
		II		4	0.2318	0.2587	0.2582	0.2592	0.2452	0.2450	0.2455
				5	0.3005	0.3182	0.3176	0.3187	0.3093	0.3090	0.3096
				6	0.3601	0.3749	0.3743	0.3755	0.3675	0.3672	0.3678
				7	0.4254	0.4370	0.4364	0.4376	0.4312	0.4309	0.4315
	5	I	3	5	0.2227	0.2360	0.2358	0.2363	0.2294	0.2292	0.2295
		II		4	0.7568	0.2528	0.2520	0.2537	0.5048	0.5012	0.5084
				5	0.2716	0.2981	0.2976	0.2987	0.2849	0.2846	0.2852
				6	0.3418	0.3577	0.3572	0.3583	0.3497	0.3495	0.3500
				7	0.3978	0.4104	0.4098	0.4109	0.4041	0.4038	0.4043
2	1	I	3	5	0.3825	0.3582	0.3555	0.3610	0.3704	0.3690	0.3718
		II		4	0.6195	0.6443	0.6407	0.6481	0.6319	0.6301	0.6338
				5	0.8458	0.8233	0.8196	0.8270	0.8346	0.8327	0.8364
				6	1.0461	1.0028	0.9988	1.0069	1.0244	1.0265	1.0224
				7	1.2689	1.2067	1.2022	1.2112	1.2378	1.2355	1.2401
	3	I	3	5	0.5712	0.5698	0.5682	0.5715	0.5705	0.5697	0.5714
		II		4	1.2445	1.1534	1.1390	1.1687	1.1990	1.1917	1.2067
				5	1.7318	1.5086	1.4923	1.5259	1.6202	1.6114	1.6294
				6	2.1173	1.8128	1.7947	1.8318	1.9650	1.9547	1.9756
				7	2.4783	2.0767	2.0609	2.0929	2.2775	2.2674	2.2875
	5	I	3	5	0.9125	0.8671	0.8633	0.8710	0.8898	0.8879	0.8917
		II		4	1.7192	1.3544	1.3319	1.3784	1.5368	1.5237	1.5503
				5	2.3935	1.8512	1.8302	1.8731	2.1224	2.1079	2.1367
				6	2.8661	2.2377	2.2156	2.2606	2.5519	2.5356	2.5679
				7	3.2881	2.6057	2.5832	2.6288	2.9469	2.9295	2.9639

**Table 8:** Lower and upper bounds for $z_{\tau :N},$ $\tau =5,\ldots ,N,$ for data sets 1 and 2 based on statistic $\Psi$ for fixed (Case I) and random sample sizes (Case II).

$\pi$	Case	*s*	$\tau$	$k=1$			$k=3$			$k=5$		
				$z_{\tau :N}$	PCI	IW	$z_{\tau :N}$	PCI	IW	$z_{\tau :N}$	PCI	IW
$0.05^{a}$	I	3	4	0.5330	(0.4600,1.6000)	1.1399	0.5330	(0.3128,1.8030)	1.4902	2.1800	(1.1395,2.9670)	1.8275
			5	0.0680	(0.3604,1.6000)	1.2395	0.5330	(0.2540,1.8030)	1.5490	1.3000	(1.0357,2.9670)	1.9313
			6	0.0680	(0.0614,1.6000)	1.5386	0.5080	(0.2074,1.8030)	1.5956	1.0660	(0.9463,2.9670)	2.0207
	II		4	0.5330	(0.4984,1.6000)	1.1016	0.6330	(0.3127,1.8030)	1.4902	2.1800	(0.8683,2.9670)	2.0986
			5	0.0680	(0.2837,1.6000)	1.3162	0.5330	(0.2540,1.8030)	1.5490	1.3000	(0.7996,2.9670)	2.1674
			6	0.0680	(0.0183,1.6000)	1.5816	0.5330	(0.2074,1.8030)	1.5955	1.0660	(0.7385,2.9670)	2.2285
$0.10^{a}$	I		4	0.5330	(0.4460,1.6000)	1.1541	0.5330	(0.2998,1.8030)	1.5031	2.1800	(1.1174,2.9670)	1.8496
			5	0.0680	(0.3415,1.6000)	1.2585	0.5330	(0.2438,1.8030)	1.5591	1.3000	(1.0168,2.9670)	1.9502
			6	0.0680	(0.0677,1.6000)	1.5323	0.5080	(0.1993,1.8030)	1.6036	1.0660	(0.9299,2.9670)	2.0371
	II		4	0.5330	(0.4753,1.6000)	1.1248	0.6330	(0.2998,1.8030)	1.5031	2.1800	(0.8538,2.9670)	2.1131
			5	0.0680	(0.2625,1.6000)	1.3375	0.5330	(0.2438,1.8030)	1.5591	1.3000	(0.7868,2.9670)	2.1802
			6	0.0680	(0.0137,1.6000)	1.5863	0.5330	(0.1993,1.8030)	1.6036	1.0660	(0.7271,2.9670)	2.2399
$0.05^{b}$	I	3	4	0.0500	(0.0$^{2}$318,0.0800)	0.0799	0.3100	(0.0$^{2}$823,0.3800)	0.3797	0.3100	(0.0$^{2}$351,0.4100)	0.4096
			5	0.0200	(0.0$^{2}$224,0.0800)	0.0799	0.0700	(0.0$^{2}$706,0.3800)	0.3799	0.1200	(0.0$^{2}$118,0.4100)	0.4098
			6	0.0100	(0.0$^{2}$157,0.0800)	0.0800	0.0400	(0.0$^{2}$623,0.3800)	0.3800	0.0800	(0.0$^{2}$107,0.4100)	0.4099
	II		4	0.0500	(0.0$^{2}$239,0.0800)	0.0800	0.3100	(0.0$^{2}$223,0.3800)	0.3798	0.3100	(0.0$^{2}$533,0.4100)	0.4099
			5	0.0200	(0.0$^{2}$124,0.0800)	0.0800	0.0700	(0.0$^{2}$706,0.3800)	0.3799	0.1200	(0.0$^{2}$450,0.4100)	0.4100
			6	0.0100	(0.0$^{2}$123,0.0800)	0.0800	0.0600	(0.0$^{2}$223,0.3800)	0.3800	0.0800	(0.0$^{2}$151,0.4100)	0.4100
$0.10^{b}$	I		4	0.0500	(0.0$^{2}$390,0.0800)	0.0799	0.3100	(0.0$^{2}$677,0.3800)	0.3798	0.3100	(0.0$^{2}$282,0.4100)	0.4097
			5	0.0200	(0.0$^{2}$224,0.0800)	0.0799	0.0700	(0.0$^{2}$561,0.3800)	0.3799	0.1200	(0.0$^{2}$250,0.4100)	0.4099
			6	0.0100	(0.0$^{2}$154,0.0800)	0.0800	0.4000	(0.0$^{2}$177,0.3800)	0.3800	0.0800	(0.0$^{2}$219,0.4100)	0.4100
	II		4	0.0500	(0.0$^{2}$457,0.0800)	0.0800	0.3100	(0.0$^{2}$177,0.3800)	0.3798	0.3100	(0.0$^{2}$408,0.4100)	0.4099
			5	0.0200	(0.0$^{2}$373,0.0800)	0.0800	0.0700	(0.0$^{2}$561,0.3800)	0.3799	0.1200	(0.0$^{2}$361,0.4100)	0.4100
			6	0.0100	(0.0$^{2}$223,0.0800)	0.0800	0.0600	(0.0$^{2}$177,0.3800)	0.3800	0.0800	(0.0$^{2}$121,0.4100)	0.4100

Notation $$0.0^{c}w=w\times 10^{-c-1}$$.

Notation *b* and *a* refer to Data sets 1 and 2, respectively

## Supplementary Information

Supplementary Information.

## Data Availability

The datasets used and analyzed during the current study available from the corresponding author on reasonable request.
