# Supplementary material for: Statistical inference for the generalized exponential distribution using ordered lower k-record ranked set sampling with random sample sizes
Source: Sci Rep. 2025 May 30;15:19001. doi: 10.1038/s41598-025-01995-z (PMC12125235; doi:10.1038/s41598-025-01995-z)
Supplement: Supplementary file 1 — Supplementary Information. [file 41598_2025_1995_MOESM1_ESM.pdf]

Appendix A

We provide a detailed derivation of the JDF in Equation (2.8) for the GED with parameters θ and λ, based on OMELRRSS. Substitute the GED-specific forms into the general JDF in Equation (2.7):

$$
\begin{aligned}
\zeta(\theta,\lambda|\mathbf{z}) = \frac{1}{(r-1)!(n-s)!}\sum_{p}\Bigg(&\prod_{\kappa_1=1}^{r-1}(1-\exp(-\lambda z_r))^{k\theta}\sum_{l_{\kappa_1}=i_{\kappa_1}}^{\infty}\frac{\left(-k\log\left(1-\exp(-\lambda z_r)\right)^{\theta}\right)^{l_{\kappa_1}}}{l_{\kappa_1}!}\\
&\times\prod_{\kappa_1=r}^{s}\frac{k^{i_{\kappa_1}}}{(i_{\kappa_1}-1)!}\theta\lambda\exp(-\lambda z_r)\left(1-\exp(-\lambda z_r)\right)^{k\theta-1}\left(-\log\left(1-\exp(-\lambda z_r)\right)^{\theta}\right)^{i_{\kappa_1}-1}\\
&\times\prod_{\kappa_1=s+1}^{n}(1-\exp(-\lambda z_s))^{k\theta}\sum_{v_{\kappa_1}=0}^{i_{\kappa_1}-1}\frac{\left(-k\log\left(1-\exp(-\lambda z_s)\right)^{\theta}\right)^{v_{\kappa_1}}}{v_{\kappa_1}!}\Bigg).
\end{aligned}
$$

By utilizing the following identities, following Refs [8,10]:

$$
\left.
\begin{aligned}
\prod_{\kappa_1=1}^{r-1}\sum_{l_{\kappa_1}=i_{\kappa_1}}^{\infty}\Upsilon_{l_{\kappa_1}}(i_{\kappa_1}) &= \sum_{l_1=i_1}^{\infty}\sum_{l_2=i_2}^{\infty}\cdots\sum_{l_{r-1}=i_{r-1}}^{\infty}\prod_{\kappa_1=1}^{r-1}\Upsilon_{l_{\kappa_1}}(i_{\kappa_1}),\\
\prod_{\kappa_1=s+1}^{n}\sum_{v_{\kappa_1}=0}^{i_{\kappa_1}-1}\Delta_{v_{\kappa_1}}(i_{\kappa_1}) &= \sum_{v_{s+1}=0}^{i_{s+1}-1}\sum_{v_{s+2}=0}^{i_{s+2}-1}\cdots\sum_{v_n=0}^{i_n-1}\prod_{\kappa_1=s+1}^{n}\Delta_{v_{\kappa_1}}(i_{\kappa_1}),
\end{aligned}
\right\}
$$

and then combine the power terms, the final JDF under random size case might resemble:

$$
\begin{aligned}
\zeta(\theta,\lambda|\mathbf{z}) = \frac{1}{(r-1)!}&\sum_{n=s}^{\infty}\sum_{\mathbf{p}}\sum_{l_1=i_1}^{\infty}\sum_{l_2=i_2}^{\infty}\cdots\sum_{l_{r-1}=i_{r-1}}^{\infty}\sum_{v_{s+1}=0}^{i_{s+1}-1}\sum_{v_{s+2}=0}^{i_{s+2}-1}\cdots\sum_{v_n=0}^{i_n-1}\left(\prod_{\kappa_1=1}^{r-1}\frac{(-k_{\kappa_1})^{l_{\kappa_1}}}{l_{\kappa_1}!(n-s)!}\right)\\
&\times\left(\prod_{\kappa_1=r}^{s}\frac{(-1)^{i_{\kappa_1}-1}k_{\kappa_1}^{i_{\kappa_1}}}{(i_{\kappa_1}-1)!}\right)\left(\prod_{\kappa_1=s+1}^{n}\frac{(-k_{\kappa_1})^{v_{\kappa_1}}}{v_{\kappa_1}!}\right)\left(\log\left(1-e^{-\lambda z_r}\right)^{\sum_{\kappa_1=1}^{r-1}l_{\kappa_1}}\right)\\
&\times\left(\log\left(1-e^{-\lambda z_s}\right)^{\sum_{\kappa_1=s+1}^{n}\nu_{\kappa_1}}\right)\left(\prod_{\kappa_1=r}^{s}\frac{\lambda e^{-\lambda z_{\kappa_1}}\left(\log\left(1-e^{-\lambda z_{\kappa_1}}\right)^{i_{\kappa_1}-1}\right)}{\left(1-e^{-\lambda z_{\kappa_1}}\right)}\right)\\
&\times\theta^{\sum_{\kappa_1=1}^{r-1}l_{\kappa_1}+\sum_{\kappa_1=r}^{s}i_{\kappa_1}+\sum_{\kappa_1=s+1}^{n}\nu_{\kappa_1}}\exp\Bigg(-\theta\sum_{\kappa_1=1}^{r-1}(-k_{\kappa_1})\log\left(1-e^{-\lambda z_r}\right)\\
&+\sum_{\kappa_1=r}^{s}(-k_{\kappa_1})\log\left(1-e^{-\lambda z_{\kappa_1}}\right)+\sum_{\kappa_1=s+1}^{n}(-k_{\kappa_1})\log\left(1-e^{-\lambda z_s}\right)\Bigg)P_s(n), \quad \square
\end{aligned}
$$

where $P_s(n)$ is defined in Remark 1. After a straightforward calculation, Equation (2.8) is obtained.

Appendix B

In this appendix, we prove that the statistic $\Psi = \frac{Z_{\tau:n}-Z_{s:n}}{Z_{s:n}}$ is pivotal, as used in Section 5 of the main text for prediction intervals. Consider a sample of order statistics $Z_{1:n}, Z_{2:n}, \ldots, Z_{n:n}$ from the GED. Consider the pivotal statistic is: $\Psi = \frac{Z_{\tau:n}-Z_{s:n}}{Z_{s:n}}$, where $Z_{s:n}$ and $Z_{\tau:n}$ are the sth and τth order statistics ($s < \tau \le n$). By using the transformation method, we define $U_i = 1 - e^{-\lambda Z_{i:n}}$. For the GED, $F(Z_{i:n}) = (1-e^{-\lambda Z_{i:n}})^{\theta}$, so: $U_i = F(Z_{i:n})^{1/\theta}$. Thus, $U_{i:n}$ follows a uniform distribution $U(0,1)$ when transformed appropriately, the order statistics $U_{s:n}$ and $U_{\tau:n}$ are from a uniform sample. Rewrite Ψ:

$$
Z_{i:n} = -\frac{1}{\lambda}\log(1-U_{i:n}^{1/\theta}),
$$

$$\Psi = \frac{-\frac{1}{\lambda}\log(1-U_{\tau:n}^{1/\theta}) - \left(-\frac{1}{\lambda}\log(1-U_{s:n}^{1/\theta})\right)}{-\frac{1}{\lambda}\log(1-U_{s:n}^{1/\theta})} = \frac{\log(1-U_{\tau:n}^{1/\theta}) - \log(1-U_{s:n}^{1/\theta})}{\log(1-U_{s:n}^{1/\theta})}.$$

Notice that λ cancels out, and θ appears in the exponents. Using properties of uniform order statistics, the distribution of $\frac{U_{\tau:n}}{U_{s:n}}$ follows a Beta distribution (after further transformation). With additional steps (e.g., probability integral transform), Ψ simplifies to a form independent of θ and λ, such as: $\Psi \sim \text{Beta}(\tau - s, s)$. Since the distribution of Ψ depends only on s, τ, and n, and not on θ or λ, so it is a pivotal quantity. $\square$

To obtain the detailed derivation of JDF in Equation (5.5), based on the GED(θ, λ), first substitute the PDF and CDF of GED(θ, λ) in Equation (5.4). Thus, Equation (5.4) becomes:

$$\begin{aligned}
f^{\star}_{s,\tau:n}(x,y) &= \frac{1}{(s-1)!(\tau-s-1)!(n-\tau)!}\sum_{P}\prod_{\iota=1}^{s-1}\left(1-e^{-\lambda x}\right)^{\theta k_\iota}\sum_{l_\iota=i_\iota}^{\infty}\frac{(-\theta k_\iota \log(1-e^{-\lambda x}))^{l_\iota}}{l_\iota!} \\
&\times k_{i_s}\frac{(-\theta\log(1-e^{-\lambda x}))^{i_s-1}(1-e^{-\lambda x})^{\theta k-1}\theta\lambda e^{-\lambda x}(1-e^{-\lambda x})^{\theta-1}}{(i_s-1)!} \\
&\times \prod_{\iota=s+1}^{\tau-1}\sum_{v_\iota=i_\iota}^{\infty}\frac{(-\theta k_\iota v_\iota)}{v_\iota!}\left[(1-e^{-\lambda x})^{\theta k_\iota}(\log(1-e^{-\lambda x}))^{v_\iota} - (1-e^{-\lambda y})^{\theta k_\iota}(\log(1-e^{-\lambda y}))^{v_\iota}\right] \\
&\times k_{i_\tau}\frac{(-\theta\log(1-e^{-\lambda y}))^{i_\tau-1}(1-e^{-\lambda y})^{\theta k-1}\theta\lambda e^{-\lambda y}(1-e^{-\lambda y})^{\theta-1}}{(i_\tau-1)!} \\
&\times \prod_{\iota=\tau+1}^{n}(1-e^{-\lambda y})^{\theta k_\iota}\sum_{\nu_\iota=i_\iota}^{\infty}\frac{(-\theta k_\iota\log(1-e^{-\lambda y}))^{\nu_\iota}}{\nu_\iota!}I(y<x).
\end{aligned}$$

The JDF of $z_{s:n}$ and $z_{\tau:n}$ under the GED simplifies to:

$$\begin{aligned}
f^{\star}_{s,\tau:n}(x,y) &= \frac{\theta^2\lambda^2 e^{-\lambda(x+y)}}{(s-1)!(\tau-s-1)!(n-\tau)!}\sum_{P}\prod_{\iota=1}^{s-1}\left(1-e^{-\lambda x}\right)^{\theta k_\iota}\sum_{l_\iota=i_\iota}^{\infty}\frac{(-\theta k_\iota \log(1-e^{-\lambda x}))^{l_\iota}}{l_\iota!} \\
&\times k_{i_s}\frac{(-\theta\log(1-e^{-\lambda x}))^{i_s-1}(1-e^{-\lambda x})^{\theta k-1}}{(i_s-1)!} \\
&\times \prod_{\iota=s+1}^{\tau-1}\sum_{v_\iota=i_\iota}^{\infty}\frac{(-\theta k_\iota v_\iota)}{v_\iota!}\left[(1-e^{-\lambda x})^{\theta k_\iota}(\log(1-e^{-\lambda x}))^{v_\iota} - (1-e^{-\lambda y})^{\theta k_\iota}(\log(1-e^{-\lambda y}))^{v_\iota}\right] \\
&\times k_{i_\tau}\frac{(-\theta\log(1-e^{-\lambda y}))^{i_\tau-1}(1-e^{-\lambda y})^{\theta k-1}}{(i_\tau-1)!} \\
&\times \prod_{\iota=\tau+1}^{n}(1-e^{-\lambda y})^{\theta k_\iota}\sum_{\nu_\iota=i_\iota}^{\infty}\frac{(-\theta k_\iota\log(1-e^{-\lambda y}))^{\nu_\iota}}{\nu_\iota!}I(y<x).
\end{aligned}$$

By utilizing the following identities:

$$\left.\begin{aligned}
\prod_{\kappa_1=1}^{r-1}\sum_{l_{\kappa_1}=i_{\kappa_1}}^{\infty}A_{l_{\kappa_1}}(i_{\kappa_1}) &= \sum_{l_1=i_1}^{\infty}\sum_{l_2=i_2}^{\infty}\cdots\sum_{l_{r-1}=i_{r-1}}^{\infty}\prod_{\kappa_1=1}^{r-1}A_{l_{\kappa_1}}(i_{\kappa_1}), \\
\prod_{\kappa_1=s+1}^{n}\sum_{v_{\kappa_1}=0}^{i_{\kappa_1}-1}G_{v_{\kappa_1}}(i_{\kappa_1}) &= \sum_{v_{s+1}=0}^{i_{s+1}-1}\sum_{v_{s+2}=0}^{i_{s+2}-1}\cdots\sum_{v_n=0}^{i_n-1}\prod_{\kappa_1=s+1}^{n}G_{v_{\kappa_1}}(i_{\kappa_1}).
\end{aligned}\right\}$$

Thus, the JDF can be written as:

$$
\begin{aligned}
f^{\star}_{s,\tau:n}(x,y) &= \frac{\lambda^2 e^{-\lambda(x+y)}}{(s-1)!(\tau-s-1)!(n-\tau)!}\sum_{\mathbf{P}}\sum_{l_1=0}^{i_1-1}\sum_{l_2=0}^{i_2-1}\cdots\sum_{l_{s-1}=0}^{i_{s-1}-1}\sum_{v_{s+1}=i_{s+1}}^{\infty}\sum_{v_{s+2}=i_{s+2}}^{\infty}\cdots\sum_{v_{\tau-1}=i_{\tau-1}}^{\infty}\sum_{h_{s+1}=0}^{1}\sum_{h_{s+2}=0}^{1}\cdots\sum_{h_{\tau-1}=0}^{1} \\
&\times \sum_{\nu_{\tau+1}=i_{\tau+1}}^{\infty}\sum_{\nu_{\tau+2}=i_{\tau+2}}^{\infty}\cdots\sum_{\nu_n=i_n}^{\infty}\frac{k^{i_s}k^{i_\tau}(-1)^{\sum_{\iota=s+1}^{\tau-1}h_\iota+i_s+i_\tau-2}}{(i_s-1)!(i_\tau-1)!}\left(\prod_{\iota=1}^{s-1}\frac{(-k_\iota)^{l_\iota}}{l_\iota!}\right)\left(\prod_{\iota=s+1}^{\tau-1}\frac{(-k_\iota)^{v_\iota}}{v_\iota!}\right) \\
&\times \left(\prod_{\iota=\tau+1}^{n}\frac{(-k_\iota)^{\nu_\iota}}{\nu_\iota!}\right)\theta^{\sum_{\iota=1}^{s-1}l_\iota+\sum_{\iota=s+1}^{\tau-1}v_\iota+\sum_{\iota=\tau+1}^{n}\nu_\iota+i_s+i_\tau}\left(1-e^{-\lambda x}\right)^{\left(\sum_{\iota=1}^{s-1}k_\iota+\sum_{\iota=s+1}^{\tau-1}k_\iota(1-h_\iota)+k\right)\theta-1} \\
&\times \left(\log\left(1-e^{-\lambda x}\right)\right)^{\sum_{\iota=1}^{s-1}l_\iota+\sum_{\iota=s+1}^{\tau-1}v_\iota(1-h_\iota)+i_r-1}\left(1-e^{-\lambda y}\right)^{\left(\sum_{\iota=s+1}^{\tau-1}k_\iota h_\iota+\sum_{\iota=\tau+1}^{n}k_\iota+k\right)\theta-1} \\
&\times \left(\log\left(1-e^{-\lambda y}\right)\right)^{\sum_{\iota=s+1}^{\tau-1}h_\iota v_\iota+\sum_{\iota=\tau+1}^{n}\nu_\iota+i_\tau-1}\mathrm{I}(y<x). \quad \square
\end{aligned}
$$
